# Xiaoyaosan exerts anxiolytic-like effects by down-regulating the TNF-α/JAK2-STAT3 pathway in the rat hippocampus

**DOI:** 10.1038/s41598-017-00496-y

**Published:** 2017-03-23

**Authors:** Xiao-Juan Li, Qing-Yu Ma, You-Ming Jiang, Xiao-Hui Bai, Zhi-Yi Yan, Qun Liu, Qiu-Xia Pan, Yue-Yun Liu, Jia-Xu Chen

**Affiliations:** School of Basic Medical Science, Beijing University of Chinese Medicine, No. 11 North Third Ring Road Chaoyang District, Beijing, 100029 China

## Abstract

Although the anxiolytic-like effects of Xiaoyaosan, a Chinese herbal formula, have been described in many previous studies, its underlying mechanism remains undefined. The cytokine tumour necrosis factor-α (TNF-α) and its closely associated janus kinase 2 (JAK2)-signal transducer and activator of transcription (STAT3) signalling pathway regulate the neuro-inflammatory response in the brain, thus participating in the development of anxiety. Our purpose was to investigate whether the anxiolytic-like effects of Xiaoyaosan are related to the TNF-α/JAK2-STAT3 pathway in the hippocampus. We examined the effects of Xiaoyaosan on behaviours exhibited in the elevated plus maze test, open field test and novelty-suppressed feeding test as well as hippocampal neuron damage and changes in the TNF-α/JAK2-STAT3 pathway in a rat model of chronic immobilization stress (CIS)-induced anxiety. Xiaoyaosan exerts anxiolytic-like effects on CIS-induced anxiety, with a significant alleviation of anxiety-like behaviours, an attenuation of hippocampal neuron damage, and a reversal of the activation of the TNF-α/JAK2-STAT3 pathway in the hippocampus that are similar to the effects of the JAK2 antagonist AG490. However, Xiaoyaosan and AG490 failed to effectively regulate apoptosis-related factors, including Bax and Caspase-3. These results suggest that Xiaoyaosan attenuates stress-induced anxiety behaviours by down-regulating the TNF-α/JAK2-STAT3 pathway in the rat hippocampus.

## Introduction

Chronic stress is thought to be a risk factor that contributes to the development of psychiatric disorders, such as anxiety and depression^1^. However, to date, the mechanism by which stress induces behavioural disorders is still unclear. Chronic exposure to stress has been shown to have a profound effect on immune function^2, 3^. Microglia, innate immune-related glial cells in the central nervous system (CNS), have typically been shown to play a central role in mediating the neuro-inflammatory effects of stress. Consequently, activated and modified microglia can release an array of inflammatory cytokines, such as interleukin-1β (IL-1β) and tumour necrosis factor-α (TNF-α), leading to the development of stress-related diseases^4, 5^. Based on accumulating evidence, stress and the neuroinflammatory cascade are involved in the pathogenesis of anxiety and depression^6, 7^. In addition, neuroinflammatory damage, particularly in the hippocampal region, is associated with the pathogenesis of anxiety and depression^8, 9^. The up-regulation of inflammatory cytokines in the CNS is understood to negatively interact with various pathways, resulting in the accumulation of neurotoxic products^10^. Recently, the Janus kinase (JAK)-signal transducer and activator of transcription (STAT) pathway, a specific signal transduction pathway, has been increasingly considered to play an important role in anxiety^11, 12^, and further studies showed that hypersecretion of TNF-α in the CNS triggered the activation of the JAK2-STAT3 signalling pathway^13^. Therefore, research on the inhibitory effects of anti-anxiety drugs on the TNF-α/JAK-STAT pathway could reveal the targets or mechanisms of action of these drugs.

Xiaoyaosan has been classically prescribed for the treatment of mental disorders for thousands of years in China. Xiaoyaosan was originally described in *Taiping Huimin Heji Jufang*, a Chinese materia medica officially compiled in the Song Dynasty of China (960-1127 AD), as a formula consisting of eight crude herbs: *Radix Bupleuri*, *Radix Paeoniae Alba*, *Radix Angelicae Sinensis*, *Rhizoma Atractylodis*, *Poria*, *Radix Glycyrrhizae*, *Herba Menthae* and *Rhizoma Zingiberis Recens*. Xiaoyaosan has been shown to be effective at treating the syndrome of liver qi stagnation and spleen deficiency, a syndrome closely related to mental illnesses, such as anxiety and depression, in traditional Chinese medicine (TCM).

As shown in our previous report, Xiaoyaosan has regulatory effects on chronic immobilization stress (CIS)-induced behavioural disorders, and we performed a series of experiments to determine the underlying mechanisms of action of Xiaoyaosan. For example, Xiaoyaosan effectively regulated hippocampal levels of brain-derived neurotrophic factor (BDNF) and its receptors and consequently helped reduce stress-induced depression^14^; Xiaoyaosan increased the key parameters of the elevated plus maze (EPM) test that are negatively associated with anxiety-like behaviour, which was reported to restore the balance of α-amino-3-hydroxy-5-methyl-4-isoxazolepropionic acid (AMPA) receptor signalling associated with rapid antidepressant responses in stress-exposed rats^15, 16^. Moreover, we found that Xiaoyaosan enhanced anxiolytic-like effects in the amygdala by regulating the corticotropin-releasing factor 1 receptor (CRF1R) signalling pathway^17^. Similarly, many parallel studies supported the ability of Xiaoyaosan to ameliorate stress-related depressive-like behaviours by suppressing the hyperactivation of the hypothalamic-pituitary-adrenal (HPA) axis^18^. Although many efforts have been focused on understanding the mechanism by which Xiaoyaosan reduces anxiety-like behaviours, few studies have shown sufficient interest in exploring the anxiolytic-like effects of Xiaoyaosan through the regulation of the immune system, particularly the neuro-inflammatory responses in the CNS. However, based on strong experimental and clinical evidence, cytokine signalling pathways in the brain are important contributors to the pathogenesis of anxiety disorders^19^. Furthermore, although the multiple potential mechanisms of action of Xiaoyaosan in producing anxiolytic-like effects may be primarily attributed to its complex combination of compounds, the crucial roles of Xiaoyaosan and its individual active compounds in immune signalling are relevant. Thus, the aim of the present study was to further clarify whether Xiaoyaosan suppresses anxiolytic-like behaviours by regulating the critical TNF-α/JAK2-STAT3 cytokine signalling pathway in the CNS.

## Results

### Effects of Xiaoyaosan on anxiety-like behaviours

We conducted a series of behavioural tests, including the EPM test, open field test (OFT) and novelty-suppressed feeding (NSF) test, to examine the effects of Xiaoyaosan on anxiety-like behaviours.

**Figure 1:**
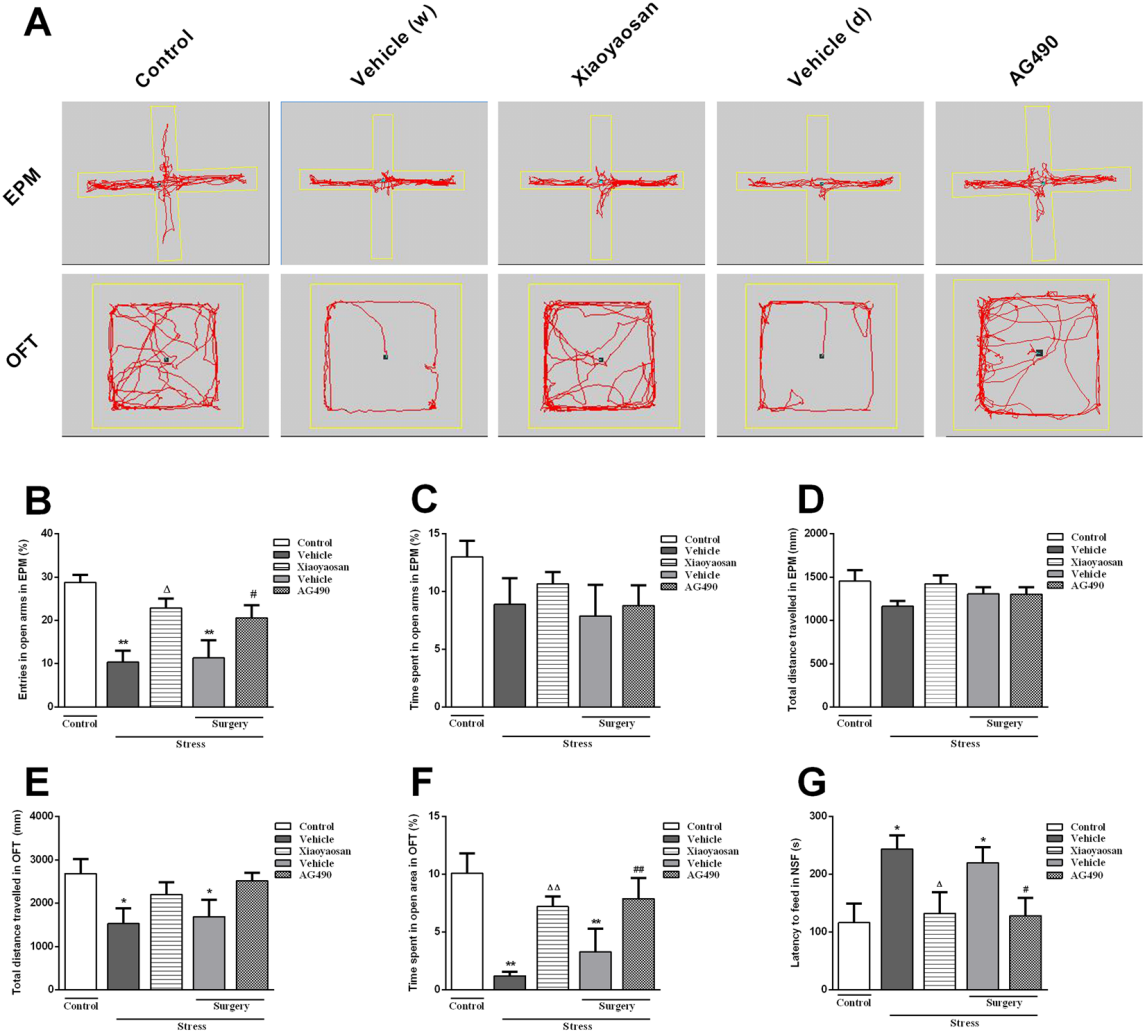
Effects of Xiaoyaosan on anxiety-like behaviours. The EPM test, OFT, and NSF test were conducted at the end of the experiment. The percentage of entries and time spent in the open arms and the total distance travelled in the EPM were measured. The total distance travelled and percentage of time spent in open area of the OFT were determined. The latency of the rats to feed within 5 min in the NSF test were recorded. (**A**) The track maps of the rats in the different groups in the EPM test and OFT. (**B**) Percentage of entries in open arms of the EPM. (**C**) Percentage of time spent in the open arms of the EPM. (**D**) Total distance travelled in the EPM. (**E**) Total distance travelled in the OFT. (**F**) Percentage of time spent in the open area of the OFT. (**G**) Latency to feed in the NSF test. Values are presented as the means ± SEM with 9–10 rats in each group. *P < 0.05 or **P < 0.01 versus the control group. ^Δ^ *P* < 0.05 or ^ΔΔ^ *P* < 0.01 versus the vehicle (distilled water) group. ^#^ *P* < 0.05 or ^##^ *P* < 0.01 versus the vehicle (dissolvent) group.

For the EPM test, as shown in Fig. 1B, the percentage of entries in the open arms was significantly lower in the vehicle (distilled water or dissolvent) groups than in the control group (*P* < 0.01). Xiaoyaosan-treated rats showed a greater percentage of entries in open arms than the vehicle (distilled water)-treated rats (*P* < 0.05). A significant increase in the percentage of entries in open arms was observed in the AG490-treated group (a JAK2-specific inhibitor) compared with that in the vehicle (dissolvent) group (*P* < 0.05). A slight increase in the percentage of time spent in open arms (Fig. 1C) was observed in the vehicle (distilled water or dissolvent) groups compared to the behaviours observed in the control group, but no significant differences were observed (*P* > 0.05). Similarly, significant differences in the total distance travelled were not observed among the 5 groups (Fig. 1D, *P* > 0.05).

For the OFT shown in Fig. 1E, the CIS-exposed rats displayed a significant decrease in the total distance travelled in the OFT. Although significant differences were not observed between the Xiaoyaosan group and the vehicle (distilled water) group or between the AG490 and the vehicle (dissolvent) groups, both the Xiaoyaosan and AG490 treatments slightly reversed the CIS-induced decrease in the total distance travelled. As shown in Fig. 1F, the vehicle (distilled water or dissolvent) or groups of CIS-exposed rats spent significantly less time in the open area than the control group (*P* < 0.01). Both the Xiaoyaosan and AG490 treatments remarkably reversed the CIS-induced decrease in the time spent in the open area (*P* < 0.01).

For the NSF test shown in Fig. 1G, CIS exposure (the vehicle-treated with distilled water or dissolvent) resulted in a significant increase in the latency to feed compared with that observed in the control group (*P* < 0.05). The increase observed in the NSF test was ameliorated by the administration of Xiaoyaosan or AG490 (*P* < 0.05).

### Effects of Xiaoyaosan on hippocampal neurons

**Figure 2:**
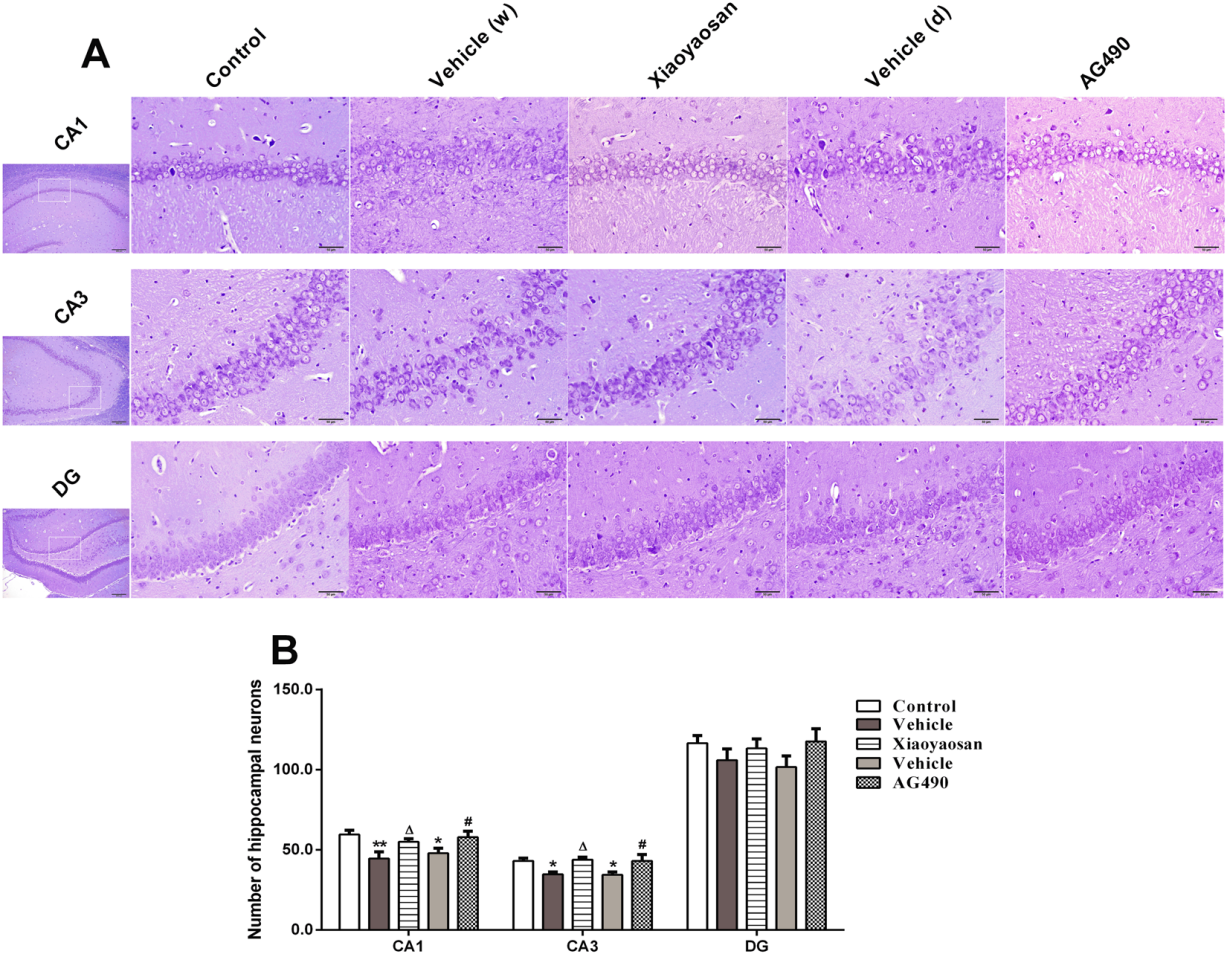
The effects of Xiaoyaosan on hippocampal neurons were measured using Cresyl violet staining. (**A**) Representative micrographs of the CA1, CA3 and DG regions of the hippocampus. The first row of micrographs was captured at low magnification under a light microscope (scale bar = 200 μm, 100 × magnification); the remaining micrographs were captured at higher magnification under a light microscope (scale bar = 50 μm, 400 × magnification). (**B**) Quantitative analysis of the numbers of Cresyl violet-stained neurons in the hippocampal CA1, CA3 and DG regions. Values are presented as the means ± SEM from 5 rats in each group. *P < 0.05 or **P < 0.01 versus the control group. ^Δ^ *P* < 0.05 versus the vehicle (distilled water) group. ^#^ *P* < 0.05 versus the vehicle (dissolvent) group.

Cresyl violet staining was performed to investigate the neuroprotective effect of Xiaoyaosan on stress-induced neuronal injury in rat hippocampal subregions, including CA1, CA3 and DG. The normal Cresyl violet-stained neurons were large, round cells with identifiable cell membranes, nuclei and discrete nucleoli, whereas neurons showing an oval-shaped or irregular cell with shrunken perikarya, small-sized and condensed nuclei or the lack of a nucleolus were counted as injured neurons (Fig. 2A). As shown in Fig. 2B, compared with the hippocampal CA1 and CA3 neurons in the control group, the numbers of normal Cresyl violet-stained cells were markedly decreased in the stressed groups treated with vehicle (distilled water or dissolvent) (*P* < 0.05 and *P* < 0.01, respectively), whereas the administration of Xiaoyaosan or AG490 reversed the neuronal injury (*P* < 0.05). Although injured neurons were also observed in the hippocampal DG region in the stressed groups treated with vehicle (distilled water or dissolvent), a significant difference in the number of Cresyl violet-stained neurons was not observed among the 5 groups (*P* > 0.05).

### Effects of Xiaoyaosan on TNF**-**α, p-JAK2 and STAT3 immunolabelling

**Figure 3:**
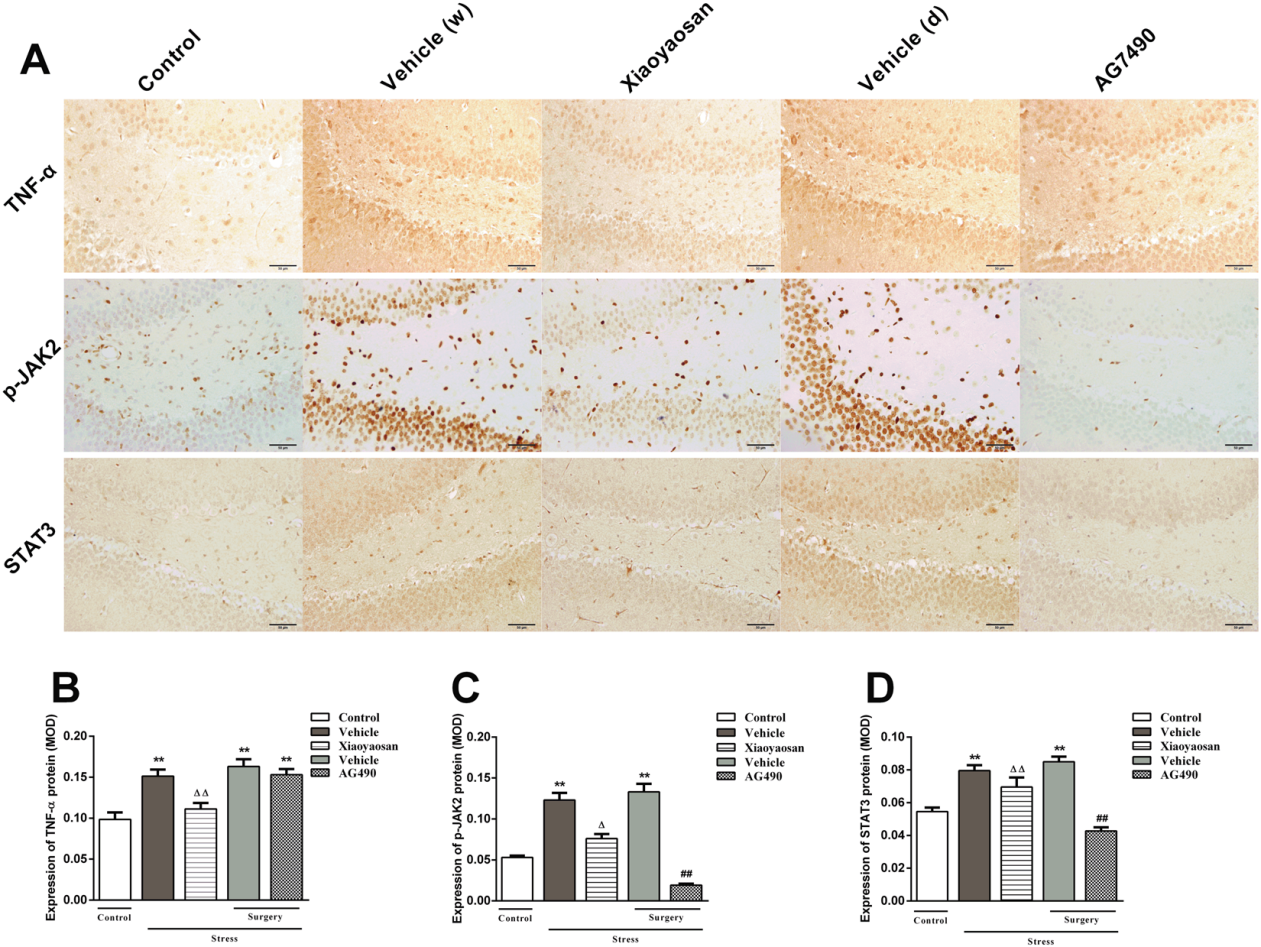
Effects of Xiaoyaosan on the expression of the TNF-α, p-JAK2 and STAT3 proteins in the rat hippocampus. (**A**) Representative micrographs of immunohistochemical staining for the TNF-α, p-JAK2 and STAT3 proteins (scale bar = 50 μm, 400 × magnification) in the hippocampal DG region. (**B**) Quantitative analysis of the TNF-α protein levels. (**C**) Quantitative analysis of the p-JAK2 protein levels. (**D**) Quantitative analysis of the STAT3 protein levels. Values are presented as the means ± SEM from 5 rats in each group. **P < 0.01 versus the control group. ^Δ^ *P* < 0.05 or ^ΔΔ^ *P* < 0.01 versus the vehicle (distilled water) group. ^##^ *P* < 0.01 versus the vehicle (dissolvent) group.

As shown in Fig. 3B, the level of TNF-α protein in the hippocampus from the vehicle (distilled water) group was markedly higher than the level in the control group (*P* < 0.01), whereas the level in the Xiaoyaosan group was significantly decreased (*P* < 0.01). Moreover, a significant increase in the level of the TNF-α protein was observed in the stressed rats from the two surgery groups compared to the level in control group (*P* < 0.01).

As shown in Fig. 3C, the level of the p-JAK2 protein in the hippocampus was markedly increased in both vehicle (distilled water and dissolvent) groups and (*P* < 0.01), whereas the levels were noticeably reduced in the AG490 and Xiaoyaosan groups (*P* < 0.01).

As shown in Fig. 3D, a significant increase in the level of the STAT3 protein was observed in the hippocampus in all of the stressed rats compared to the level in the control group, with the exception of the animals treated with AG490 (*P* < 0.01). The increased expression of the STAT3 protein in the vehicle (dissolvent) group was obviously reduced by AG490 (*P* < 0.01). However, significant differences in the expression of the STAT3 protein were not measured between the vehicle (distilled water) and Xiaoyaosan groups (*P* > 0.05).

### Effects of Xiaoyaosan on serum and hippocampal levels of TNF-α

**Figure 4:**
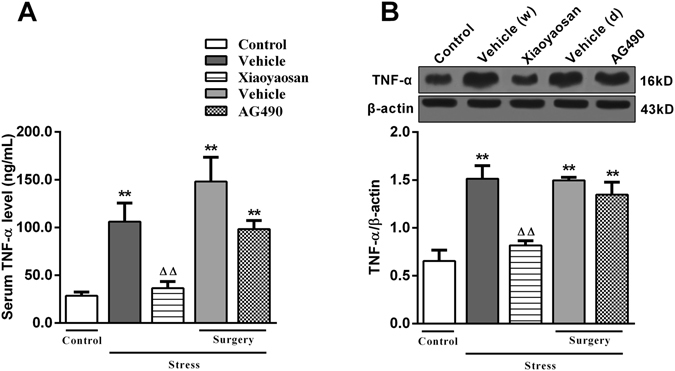
Effects of Xiaoyaosan on the serum TNF-α levels and hippocampal levels of the TNF-α protein. (**A**) Serum TNF-α levels. (**B**) Expression of the TNF-α protein in the rat hippocampus. Values are presented as the means ± SEM from 10 rats in each group for the determination of the serum TNF-α levels and 3–4 rats for the expression of the TNF-α protein in the hippocampus. **P < 0.01 versus the control group. ^ΔΔ^ *P* < 0.01 versus the vehicle (distilled water) group.

As shown in Fig. 4A, the serum TNF-α levels were significantly higher in rats exposed to stress (treated with distilled water) than in the control group, and the Xiaoyaosan treatment reduced the levels (*P* < 0.01). The stressed rats in the surgery group (treated with dissolvent) exhibited increases serum TNF-α levels compared to the control group (*P* < 0.01).

Similarly, as shown in Fig. 4B, the expression of the TNF-α protein in the hippocampus was remarkably enhanced when the rats were subjected to 14-day CIS, which was significantly reversed by the administration of Xiaoyaosan (*P* < 0.01). In contrast, the administration of AG490 failed to effectively reverse the excessive expression of the TNF-α protein in the hippocampus (*P* > 0.05).

### Effects of Xiaoyaosan on the JAK2-STAT3 signalling pathway in the rat hippocampus

**Figure 5:**
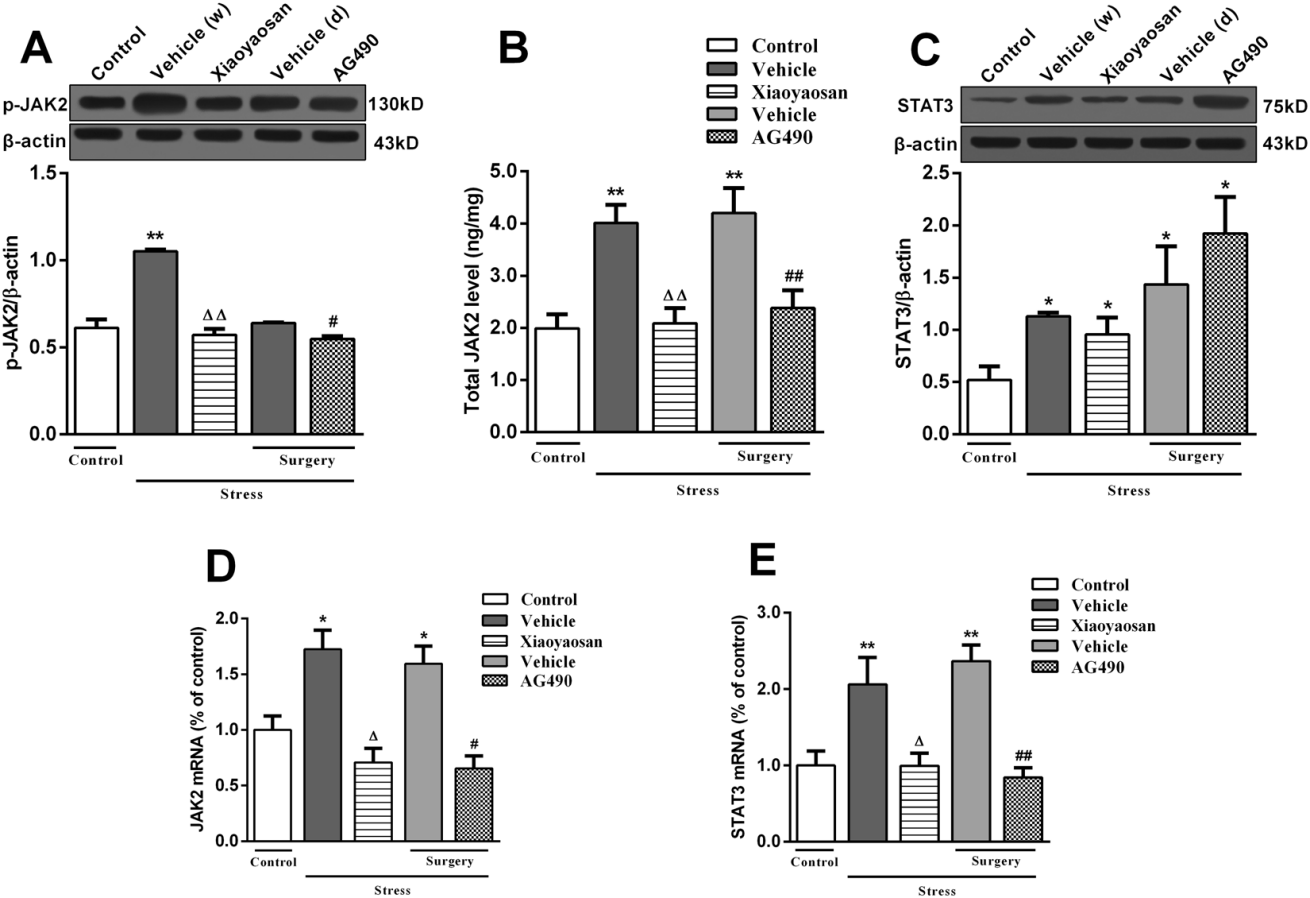
Effects of Xiaoyaosan on the JAK2-STAT3 signalling pathway in the rat hippocampus. (**A**) Expression of the p-JAK2 protein in the rat hippocampus. (**B**) Total JAK2 levels in the rat hippocampus. (**C**) Expression of the STAT3 protein in the rat hippocampus. (**D**) Expression of the JAK2 mRNA in the rat hippocampus. (**E**) Expression of the STAT3 mRNA in the rat hippocampus. Values are presented as the means ± SEM from 3–4 rats per group for determination of the expression of the p-JAK2 and STAT3 proteins, from 6 rats per group for the determination of total JAK2 levels, and from 5 rats per group for the determination of the expression of JAK2 and STAT3 mRNA in the rat hippocampus. *P < 0.05 or **P < 0.01 versus the control group. ^Δ^ *P* < 0.05 or ^ΔΔ^ *P* < 0.01 versus the vehicle (distilled water) group. ^#^ *P* < 0.05 or ^##^ *P* < 0.01 versus the vehicle (dissolvent) group.

We examined the activity of proteins in the JAK2-STAT3 signalling pathway and showed that the levels of the phosphorylated JAK2 protein and mRNA in the hippocampus were increased in the vehicle (distilled water) group compared with the control group, and these levels were attenuated by the Xiaoyaosan treatment (*P* < 0.05 and *P* < 0.01, respectively, Fig. 5A and D). Consistent with the ability of Xiaoyaosan to block JAK2 activity, the AG490 treatment decreased the levels of the phosphorylated JAK2 protein and mRNA compared with those in the vehicle (dissolvent) group (*P* < 0.05, Fig. 5A and D).

As shown in Fig. 5B, the level of total JAK2 in the hippocampus was significantly higher in rats exposed to stress (treated with distilled water) than in the control group, and the increase was attenuated by the Xiaoyaosan treatment (*P* < 0.01). Similarly, the stressed rats in the surgery group (treated with dissolvent) had significantly higher levels of total JAK2 than the control group, and the AG490 treatment significantly reduced this effect (*P* < 0.01).

The CIS procedure significantly increased the expression of the STAT3 protein and mRNA in the hippocampus (*P* < 0.05 and *P* < 0.01, respectively, Fig. 5C and E). The increase in the levels of the STAT3 mRNA was significantly attenuated by the Xiaoyaosan and AG490 treatments (*P* < 0.05 and *P* < 0.01, respectively, Fig. 5E). However, neither Xiaoyaosan nor AG490 reversed the increased expression of the STAT3 protein (*P* > 0.05, Fig. 5C).

### Effects of Xiaoyaosan on the expression of the Bcl-2, Bax and Caspase-3 proteins and mRNAs in the rat hippocampus

**Figure 6:**
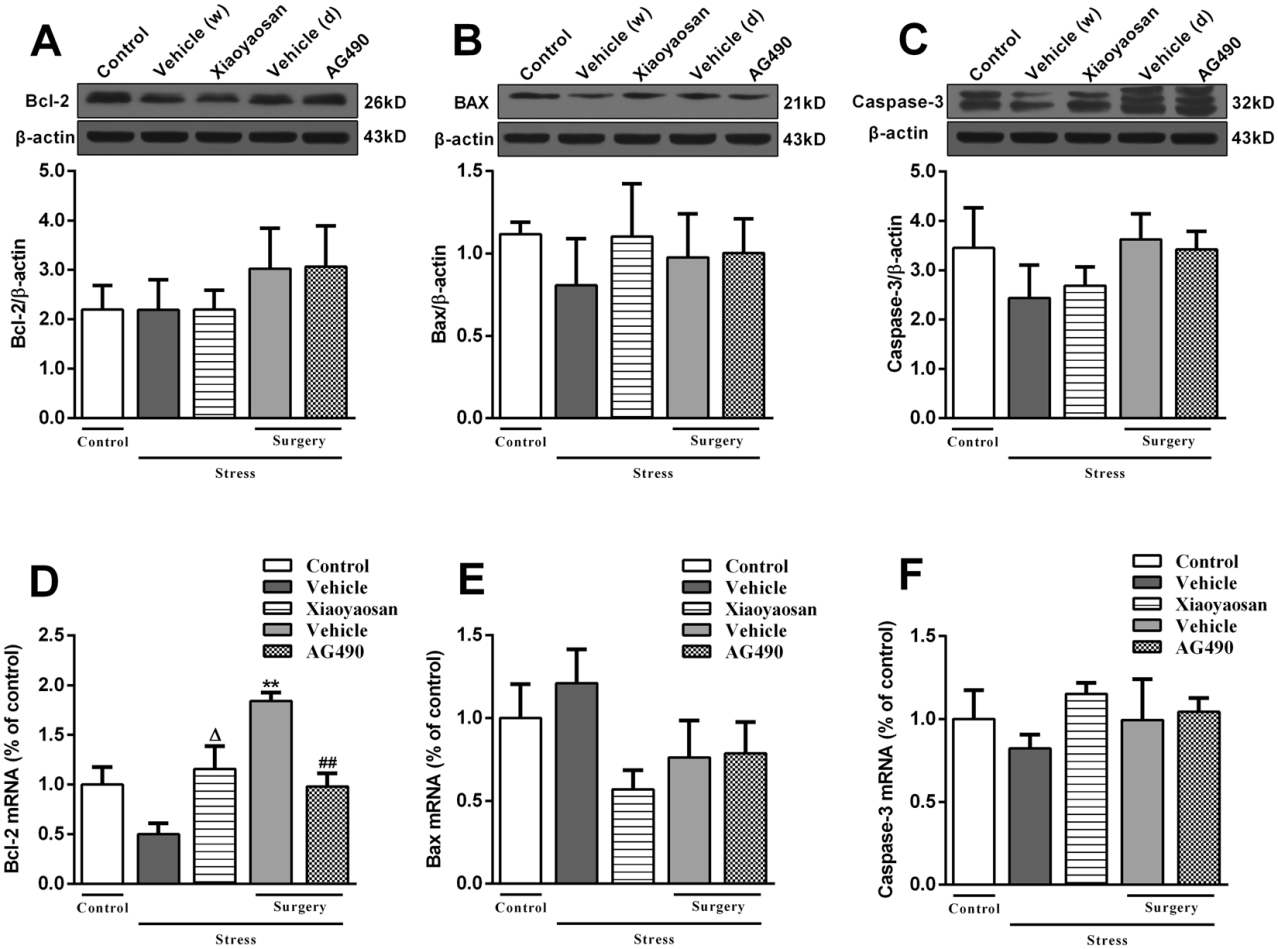
Effects of Xiaoyaosan on the expression of the Bcl-2, Bax and Caspase-3 proteins and mRNAs in the rat hippocampus. (**A**) Expression of the Bcl-2 protein in the rat hippocampus. (**B**) Expression of the Bax protein in the rat hippocampus. (**C**) Expression of the Caspase-3 protein in the rat hippocampus. (**D**) Expression of the Bcl-2 mRNA in the rat hippocampus. (**E**) Expression of the Bax mRNA in the rat hippocampus. (**F**) Expression of the Caspase-3 mRNA in the rat hippocampus. Values are presented as the means ± SEM from 3–4 rats per group for the determination of the expression of the Bcl-2, Bax and Caspase-3 proteins and from 5 rats per group for the measurement of mRNA expression in the rat hippocampus. **P < 0.01 versus the control group. ^Δ^ *P* < 0.05 versus the vehicle (distilled water) group. ^##^ *P* < 0.01 versus the vehicle (dissolvent) group.

The expression of the Bcl-2 mRNA in the Xiaoyaosan-treated group was higher than the level in the vehicle (distilled water) group (*P* < 0.05, Fig. 6D). Interestingly, stressed rats in the surgery group (treated with dissolvent) exhibited a significant increase in the expression of the Bcl-2 mRNA compared with that in the control group, and the AG490 treatment significantly reduced the expression (*P* < 0.01, Fig. 6D). However, the CIS procedure and Xiaoyaosan and AG490 treatments did not alter the expression of the Bcl-2 protein (*P* > 0.05, Fig. 6A). Moreover, the expression of the Bax and Caspase-3 proteins and mRNAs was not significantly different among the groups (*P* > 0.05, Fig. 6B,C,E and F).

## Discussion

The purpose of the present study was to investigate the anxiolytic effects of Xiaoyaosan on rats exposed to 14 days of CIS and to explore the potential mechanism underlying these anti-anxiety effects. Xiaoyaosan significantly improved the anxiety-like behaviours of stressed rats. More importantly, exposure to CIS for 14 consecutive days induced hippocampal neuron damage and triggered the release of TNF-α and the activation of the JAK2-STAT3 signalling pathway in the hippocampus, which were reversed by treatments with Xiaoyaosan or a JAK2-specific inhibitor (AG490). Furthermore, Xiaoyaosan and AG490 failed to effectively regulate the levels of apoptosis-related factors, including Bax and Caspase-3 in the 14-day CIS rat model.

Chronic stress is known to be risk factor for psychiatric disorders, and stress-induced animal models of mood disorders have therefore been widely investigated^20, 21^. Since it effectively induces the pathophysiology of anxiety and depression, chronic restraint or immobilization stress is considered an appropriate paradigm for imitating psychiatric-related illnesses in rodents^22^. Generally, anxiety-related tasks for an animal are based on natural conflicts between approach and avoidance, providing an analogy to human anxiety symptoms^23^. Currently, the widely used assays for anxiety-related behaviours include the EPM, the elevated zero-maze, the OFT, and the operant Vogel thirsty-lick conflict test^24^. In this study, stressed rats treated with vehicle (distilled water or dissolvent) showed increased anxiety-like behaviours, such as significant decreases in time spent in the open area of the OFT and increased latency to feed in NSF. Additionally, these rats also displayed a reduction in the total distance travelled in the OFT, indicating less exploration of a novel environment. However, for the EPM, there was a decreased percentage of entries into the open arms that was statistically significant. A similar trend towards less time spent in the open arms was also found, but the difference was not significant. The percentage of entries and time spent in the open arms are widely used for the evaluation of anxiety-like behaviour in rodents in the EPM^25, 26^, thus the results of EPM showed less evidence for stress-induced anxiety-like behaviour. Notably, it is not a coincidental phenomenon that there were differences in the outcomes of different behaviour tests within the same study^27, 28^. Indeed, in addition to commonly considered factors such as test protocol, laboratory environment and genetic background^29^, a review of previous findings produced a primary reason for such discrepancy that was little correlation among anxiety-related behaviours measured in different tests^30^. Therefore, even though the EPM did not provide sufficient results, the results of the OFT and NSF together corroborated the idea that exposure to CIS caused rats to enter an anxious emotional state.

Recently, extensive communication between the immune system and CNS has been clearly observed in the onset and development of neuropsychiatric disorders, in which cytokines released by central and peripheral immune cells play a critical role^12, 19^. According to previous research, communication is generalized as follows: psychiatric illnesses, including depression and anxiety, induce a disturbance in cytokine expression; antidepressants or anti-anxiety agents change the abnormal cytokines levels; high cytokines levels lead to the development and progression of depressive and anxiety disorders; cytokine inhibitor therapy contributes to the improvement of depressive and anxiety symptoms. TNF-α, one of the major pro-inflammatory cytokines, has been advocated to be a key regulator of this communication^31^. TNF-α levels have consistently been shown to be elevated in patients with anxiety disorder and in animals with anxiety-like behaviours. For example, based on accumulating evidence, a significant increase in the TNF-α levels is observed in patients with generalized anxiety disorder^32^. Similarly, parallels clearly exist in animals, where the serum TNF-α level is increased, and the deletion of TNF-α receptors reduces anxiety-like behaviours^33, 34^. These features were replicated in our study, as rats treated with vehicle (distilled water, or dissolvent) or AG490 showed increased serum and hippocampal levels of TNF-α compared with the control group. However, some contradictory results showed no changes or reduced TNF-α expression^35, 36^. One reason for this discrepancy might be attributed to the duration and variables of stress. It has been shown that immune suppression is common in the face of chronic and persistent stress, whereas immune activation occurs when stress is acute and short-lived^36^.

The JAK-STAT signalling pathway efficiently regulates gene expression primarily through two families of proteins: JAKs and STATs. Interestingly, cytokines are one of the most important factors that activate JAKs to subsequently phosphorylate STATs that translocate to the nucleus, bind to DNA and regulates transcription^37^. In the CNS, the JAK2-STAT3 signalling pathway is mainly associated with brain inflammation processes and the development and survival of neuronal/glial cells. Many studies have showed more interest in this topic. For example, activated STAT3 was observed in many cells in the CNS, and it participates in the neuronal development and neural regeneration^38^. Moreover, both JAK2 and STAT3 regulate hippocampal synaptic plasticity, which is closely related to learning and memory^11^. Consequently, increasing importance has been attached to the role of JAK2-STAT3 signalling in CNS disorders, including Alzheimer’s disease, depression and anxiety. More importantly, the significance of the role of the JAK2-STAT3 pathway in anxiety has prompted researchers to designate this pathway as a promising new drug target for mental illness because of its close correlation with the immune system in the CNS^12^. Although the role of the activated JAK2-STAT3 pathway in the release of specific cytokines has not yet been fully elucidated, accumulating evidence suggests that TNF-α activates the JAK2-STAT3 signalling pathway^13, 39^. Conversely, JAK2 and STAT3 phosphorylation are limited by the deletion of TNF-α^40^, indicating that TNF-α reliably plays a pivotal role in the activation of JAK/STAT signalling pathway. As shown in the present study, rats exposed to stress that were treated with vehicle (distilled water or dissolvent) showed elevated levels of the p-JAK2 and total JAK2 proteins and the JAK2 mRNA in the hippocampus compared with the control, subsequently increasing the expression of the STAT3 protein and mRNA. Thus, the excess release of TNF-α in the hippocampus activated the JAK2-STAT3 signalling pathway that is closely associated with anxiety-like behaviours. These findings are consistent with several previous reports. In contrast, we used a bilateral intracerebroventricular microinjection of AG490, a JAK2-specific inhibitor, to observe the role of the JAK2-STAT3 signalling pathway in CIS-induced anxiety-like behaviours in this study. In fact, AG490 has been reported to inhibit constitutively activated JAK2 in several cell types, such as eosinophils, cardiac myocytes, and vascular smooth muscle cells^41^. Consistent with these reports, AG490 inhibited the activation of JAK2 in hippocampus stimulated by high TNF-α levels; the activation of the downstream STAT3 protein was slightly reduced, indicating that AG490 had a potent anxiolytic-like effect.

The function of the activated JAK-STAT pathway is to regulate the transcription of various genes, including genes involved in apoptosis, cell growth and differentiation^42^. As shown in several previous studies, the JAK2-STAT3 signalling pathway regulates apoptosis-related genes, such as c-Caspase, Bcl-2, Bcl-x, Bax and Caspase-3^43, 44^. Additionally, one of the primary mechanisms responsible for the behavioural impairments induced by exposure to chronic stress may be related to excessive apoptosis^45, 46^. Specifically, different types of chronic stress have been shown to induce apoptosis in the hippocampus^47^. However, unlike the reports described above, significant differences in the regulation of apoptosis-related genes and proteins, including Bax and Caspase-3, were not observed in our study. This discrepancy has several possible explanations. Firstly, AG490 has been shown to prevent anti-apoptotic effect *in vivo* ^48, 49^, suggesting that the JAK2-STAT3 pathway may not have regulated apoptosis-related genes. Additionally, phosphorylated STAT3 promotes the survival of U266 myeloma cells mainly by up-regulating Bcl-XL, an anti-apoptotic protein^50^. Secondly, although the action of the JAK-STAT pathway in the regulation of apoptosis has attracted researchers’ attention, its important roles in the transcription of non-apoptotic genes that impaired hippocampal neurons were explored in recent studies. As shown in many studies, STAT3 is a crucial transcription factor involved in the up-regulation of a number of inflammatory genes, such as IL-10 and IL-6^13, 51^, suggesting that other factors are activated by JAK2-STAT3 pathway to promote impairments in the hippocampus.

Xiaoyaosan is a classic traditional Chinese herbal medicine that has been widely used to treat mental disorders in China for centuries. As more people have focused on complementary and alternative medicines (CAM), Xiaoyaosan is gradually being recognized by clinical researchers as a safe and effective prescription for the treatment of depressive and anxious disorders^52^. Recent preclinical findings also have shown the reliable antidepressant and anxiolytic effects of Xiaoyaosan by summarizing its biochemical, neurochemical, and behavioural data^53, 54^. Furthermore, increasing numbers of researchers have devoted their attention to clarifying the potential validity of the antidepressant and anxiolytic properties of this prescription. However, Xiaoyaosan consists of several Chinese herbs, and its composition is complex. Several groups of bioactive compounds have been isolated from Xiaoyaosan, including terpenoids (e.g., paeoniflorin from *Radix Paeoniae Alba*), phenolic compounds (e.g., 6-gingerol isolated from *Rhizoma Zingiberis Recens*), sterols (e.g., bupleurmol and α-spinasterol from *Radix Bupleuri* and hexahydrothymol from *Herba Menthae*), esters (e.g., ligustilide from *Radix Angelicae Sinensis* and atractylenolide III from *Rhizoma Atractylodis Macrocephalae*), acids (e.g., ferulic acid from *Radix Angelicae Sinensis* and glycyrrhizic acid from *Radix Glycyrrhizae*), polysaccharides (e.g., β-pachyman from *Poria*), and saponins (e.g., saikosaponins A, C, and D from *Radix Bupleuri*)^55^. Similarly, our team has previously determined the composition of Xiaoyaosan using HPLC-LTQ-Orbitrap-MS (high-performance liquid chromatography/linear ion trap-Orbitrap mass spectrometry) and identified eight of the main compounds, including paeoniflorin, liquiritin, glycyrrhizic acid, ferulic acid, saikosaponins A and C, curcumin, and *Poria cocos* alcohol, in Xiaoyaosan samples^56^. Based on these results, the complicated compounds contained in Xiaoyaosan may be responsible for its anxiolytic effects and different underlying mechanisms. Notably, although most of the different compounds showed noticeable anti-depressive and anxiolytic effects or neuroprotective effects, they tended to have partial immunomodulatory effects. For example, the anxiolytic effects of curcumin have been reported to be associated with its effects on reducing the levels of inflammatory cytokines, including TNF-α and IL-6, as well as their associated signalling pathways, such as the JAK-STAT and nuclear factor-κB (NF-κB) pathways^57, 58^. As shown in a previous study, α-spinasterol displaces [^3^H] resiniferatoxin from the transient receptor potential vanilloid 1 receptor (TRPV1), a potential anxiety factor, in spinal cord membranes, suggesting that its anxiolytic-like effects may be mediated through inhibition of the TRPV1 receptor^59, 60^. Meanwhile, α-spinasterol treatments directly and completely inhibit the production or release of pro-inflammatory cytokines, such as TNF-α and IL-1β, in mice injected with lipopolysaccharide (LPS)^61^. Mice administered ferulic acid showed a greater duration of time spent in the open arm and a lower number of entries in the closed arm of the EPM, indicating a significant anxiolytic effect^62^. Moreover, ferulic acid acts as a powerful antioxidant agent that exerts anti-inflammatory and neuroprotective effects^63^. In present study, Xiaoyaosan treatment significantly inhibited TNF-α release in the serum and hippocampus and the activation of JAK2-STAT3 signalling pathway in rats after exposure to CIS. Thus, the anxiolytic-like effects of Xiaoyaosan may be partially attributed to the inhibition of the TNF-α/JAK2-STAT3 pathway. However, because it is composed of multiple ingredients, the compounds in Xiaoyaosan that play key roles in ameliorating anxiety are still unknown, and more investigations will be performed in the future.

## Conclusions

The present study provides evidence for the anxiolytic-like effects of Xiaoyaosan in rats exposed to 14 consecutive days of CIS. The findings provide further support for our hypothesized mechanisms underlying the anxiolytic effects of Xiaoyaosan in the hippocampus, which involve the TNF-α/JAK2-STAT3 pathway. These findings not only help us understand the actions of Xiaoyaosan, which may inhibit the TNF-α/JAK2-STAT3 pathway in the hippocampus, but also provide new evidence for the clinical use of Xiaoyaosan as a treatment for anxiety.

## Methods

### Animals

Healthy adult male Sprague-Dawley rats (SCXK 2012-0001) with a body weight of 180–200 g were purchased from Vital River Laboratory Animal Technology Limited Company (Beijing, China) and were maintained in a temperature- and humidity-controlled room with a 12 h light-dark cycle. After one week of adaptation, the rats were randomly assigned to 5 groups of 12 rats per group: control group, vehicle (distilled water) group, Xiaoyaosan-treated group, vehicle (dissolvent) group and AG490-treated group. All animal experiments were approved by the Institutional Animal Care and Use Committee at Beijing University of Chinese Medicine and conformed to the animal welfare guidelines (BUCM-4-2013101501-4001). The experimental protocols applied in this study were performed in accordance with the approved guidelines.

### CIS procedure

The CIS procedure was conducted as previously described^64^. Briefly, the rats in all groups except for the control group were exposed to a homemade apparatus that was designed to immobilize the rats. The apparatus was composed of wooden double-binding platforms, including a lower platform (20 cm long, 10 cm wide and 2.8 cm thick) and an upper platform (22 cm long, a maximum of 6.6 cm wide and 2.8 cm thick). A small frame suitable for immobilizing a rat head was located in front of the upper platform. In addition, 2 pieces of soft, adjustable bands were available for fixing the rat neck and waist; these bands surrounded each side of the upper platform. The rats were immobilized in the apparatus for 3 h per day, without free access to food or water, and CIS was performed for 14 consecutive days from the beginning to the end of the experiment.

### Surgical procedure and intracerebroventricular microinjection

After one week of adaption, the rats in the vehicle-treated (dissolvent) and AG490-treated groups were anesthetized with 10% chloral hydrate (0.35–0.4 g/kg, i.p.) and fixed in a stereotaxic apparatus with a pair of ear bars and an incisor bar. A small incision was made to expose the skull, and the bregma was labelled to orient the coordinates. Guide cannulas (RWD Life Science, Shenzhen, China) were placed bilaterally in the lateral ventricles to enable the intracerebroventricular (i.c.v.) injections. The coordinates of the lateral ventricle were 0.92 mm anteroposterior (AP), 1.5 mm mediolateral (ML), and 3.5 mm dorsoventral (DV) to the bregma. The cannulas were secured in the skull with dental cement and three stainless steel screws. Then, the skull was covered with a dust cap to prevent clotting and dust accumulation. The rats were immediately removed from the stereotaxic apparatus and placed in an incubator to maintain their basal body temperature after surgery. Finally, the animals were intramuscularly injected with ketoprofen (10 mg/kg/day for 3 days) to reduce pain and were allowed to recover for one week.

**Figure 7:**
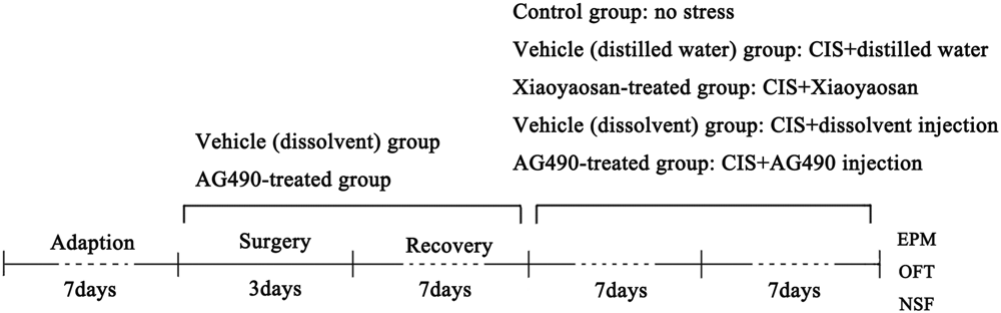
Experimental design. Prior to the experiment, the animals were allowed a 7-day adaptation period. Rats in the vehicle (dissolvent) group and AG490-treated group underwent stereotaxic surgery over 3 days and were provided a 7-day recovery period. Then, the rats in all groups, except for the control group, received a daily 3-h CIS procedure and were administered the appropriate drug for 14 consecutive days. The EPM test, OFT, and NSF test were conducted at the end of the experiment.

The i.c.v. microinjection was carefully and slowly performed using a polyethylene tube connected to a microsyringe (Hamilton, Reno, USA) on one end and the i.c.v. guide cannulas on the other. Additionally, the microinjection tube was left in place for longer than 1 min until the injected liquid had completely diffused into each of the lateral ventricles. This procedure was performed 30 min before daily immobilization stress and was performed for 14 consecutive days. The experimental design is shown in detail in Fig. 7.

### Preparation of Xiaoyaosan

Xiaoyaosan consists of eight herbs at a ratio of 3:3:3:3:3:1.5:1:1: *Radix Bupleuri* (root of *Bupleurum chinensis* DC.), *Radix Paeoniae Alba* (root of *Paeonia lactiflora* Pall.), *Radix Angelicae Sinensis* (root of *Angelica sinensis* (Oliv.) Diels), *Rhizoma Atractylodis* (root and rhizome of *Atractylodes lancea* (Thunb.) DC.), *Poria* (fungus nucleus of *Poria cocos* (Schw.) Wolf), *Radix Glycyrrhizae* (root and rhizome of *Glycyrrhiza uralensis* Fisch.), *Herba Menthae* (aboveground portions of *Mentha haplocalyx* Briq.), and *Rhizoma Zingiberis Recens* (fresh root and rhizome of *Zingiber officinale* Rosc.). All of the raw herbs were purchased from Beijing Tongrentang Group Co., Ltd. and processed into a dry extract in the China-Japan Friendship Hospital (Beijing, China) in accordance with the Regulation on Processing of Traditional Chinese Medical Herbal Pieces of Beijing. The process used to produce the dry extract of Xiaoyaosan has previously been described in detail^64^. Meanwhile, we have previously identified 8 compounds from Xiaoyaosan samples using an HPLC-LTQ-Orbitrap-MS eluted system, which matched the corresponding peaks in Xiaoyaosan^56^.

### Drug administration

The Xiaoyaosan dry extract was dissolved in distilled water and administered by gavage at a dose of 3.854 g/kg/d, 0.1 mL/kg bodyweight, which was proven to be effective in our previous studies^15, 17^. The vehicle (distilled water) group was administered 0.1 mL of distilled water/kg bodyweight. AG490 (Sigma-Aldrich, St. Louis, MO, USA) was dissolved in 0.9% pyrogen-free PBS with 25% Cremophor EL (Sigma-Aldrich, St. Louis, MO, USA) and administered through bilateral i.c.v. microinjections at a final concentration of 5 mM^65, 66^, 2 μL per lateral ventricle. The vehicle (dissolvent) group received an equal volume of the dissolvent for AG490.

### Behavioural testing

#### EPM test

The EPM apparatus consists of two open arms (45 × 15 cm), two closed arms (45 × 15 × 30 cm) and a central platform (15 × 15 cm) located 50 cm above the floor. Uncontrolled activity was monitored and recorded with a video camera. The test was performed as described below. Firstly, all of rats were moved to an observation room 30 min before the test to allow them to acclimate to the new environment. Then, each rat was gently placed in the central platform of the maze facing one of the closed arms and allowed to move freely for 5 min. The variables were measured according to our previous study, including the total distance travelled, number of arm entries, and the time spent in the arms; the percentage of time spent and number of entries in the open arms were calculated. All of the variables were analysed by Observer 5.0 software (Noldus, Netherlands) and EthoVision 3.0 software (Noldus, Netherlands).

#### OFT

The OFT was performed in a wooden open field box (125 × 125 × 50 cm) with a central zone (75 × 75 cm). All rats were placed in the testing room prior to the test to allow them to acclimate to the new environment. Then, a rat was gently placed in the centre of the apparatus and allowed to freely explore the area for 5 min. The total distance travelled and the percentage of time spent in the open area (the time spent in the open area vs the total duration) were recorded and calculated. All of the variables were analysed using Observer 5.0 software (Noldus, Netherlands) and EthoVision 3.0 software (Noldus, Netherlands).

#### NSF test

The NSF test was performed using previously described methods^17^. Briefly, the NSF apparatus consisted of a 50 × 50 × 40 cm white plastic box. Twenty-four hours prior to behavioural testing, all food was removed from the rodent’s home cage. Then, a single pellet of normal food was placed on a white paper platform positioned in the centre of the apparatus. The rats were individually placed in the corner of the plastic box, and the time rats began to chew the food pellets within 5 min was recorded as the latency to feed in a novel environment.

### Cresyl violet staining and immunohistochemical analysis

After the animals were fixed with 4% paraformaldehyde via transcardial perfusion, the brain specimens were embedded in paraffin and sliced into 5-μm thick coronal sections using a semi-motorized rotatory microtome (Leica, Wetzlar, Germany). The sections were stained with Cresyl violet to investigate the number of normal neurons with visible cell membranes, nuclei, and nucleoli in the hippocampal CA1, CA3 and DG regions.

For the immunohistochemical analysis, the slices were first immersed in a citric acid buffer solution (pH 6.0) and subsequently heated for 15 min to retrieve the antigens. Then, the sections were rinsed with 0.1 M PBS and incubated in a solution of 3% hydrogen peroxide for 10 min at room temperature to quench the endogenous peroxidases. Non-specific staining was blocked with 5% normal goat serum for 90 min at room temperature. The sections were incubated with the primary antibodies (anti-rabbit TNF-α, Abcam, San Francisco, USA, diluted 1:2,000; anti-rabbit p-JAK2 (PY1007/1008), Epitomics, Burlingame, USA, diluted 1:200; and anti-rabbit STAT3, Epitomics, Burlingame, USA, diluted 1:100) diluted in 0.1 M PBS containing 5% normal goat serum overnight at 4 °C. Afterwards, the sections were rinsed again and incubated with the corresponding horseradish peroxidase (HRP)-conjugated secondary antibodies (anti-mouse, ZSGB-BIO, Beijing, China, diluted 1:2,000; and anti-rabbit, ZSGB-BIO, Beijing, China, 1:2,000) in 0.1 M PBS containing 5% normal goat serum for 2 h at room temperature, and then rinsed again. Subsequently, the sections were incubated in a 3,3′-diaminobenzidine tetrahydrochloride (DAB) solution (ZSGB-BIO, Beijing, China) for 5–15 min at room temperature. The sections were mounted on gelatine-coated glass slides and the morphological changes were observed under a light microscope (BX53, Olympus Co., Tokyo, Japan). For quantification, two investigators who were blinded to the treatments counted the number of normal Cresyl violet-stained neurons, which were defined as large cells with identifiable cell membranes, nuclei and discrete nucleoli. The images of the immunohistochemical staining were analysed with Image-Pro Plus 6.0 software to obtain the mean optical density.

### Enzyme-linked immunosorbent assay (ELISA)

The hippocampal total JAK2 levels and serum TNF-α levels were determined with commercial ELISA kits (total JAK2, Cloud-Clone Corp., Wuhan, China; TNF-α, Multi Sciences, Hangzhou, China) according to the manufacturer’s instructions. Briefly, 100 µL of each standard or sample were pipetted into 96-well plates coated with primary antibodies and incubated on a plate shaker at room temperature. After several washes, HRP-conjugated streptavidin was added to all wells and incubated again. Then, the wells were repeatedly washed again. Subsequently, the 3,3′,5,5′-tetramethylbenzidine substrate was pipetted into the walls to generate a yellow colour that was stopped by an addition of stop solution. The mean optical density was determined using the Multiskan™ GO (Thermo Fisher Scientific, Waltham, USA) Detector system at a wavelength of 450 nm.

### Western blotting analysis

The expression of the TNF-α, p-JAK2, STAT3, Bcl-2, Bax and Caspase-3 proteins in the hippocampus was detected by Western blotting. The hippocampal tissues were homogenized and the protein-containing supernatants were separated and collected. Protein concentrations were determined using a bicinchoninic acid (BCA) kit (Thermo Fisher Scientific, Waltham, USA). Proteins were separated on 10% or 12% SDS-PAGE gels and then transferred onto polyvinylidene difluoride membranes. After blocking with 5% defatted milk for 1–3 h, the membranes were incubated with primary antibodies (anti-mouse TNF-α, Abcam, San Francisco, USA, diluted 1:500; anti-rabbit p-JAK2, Abcam, San Francisco, USA, diluted 1:1,000; anti-rabbit STAT3, Abcam, San Francisco, USA, diluted 1:1,000; anti-rabbit Bcl-2, Abcam, San Francisco, USA, diluted 1:1,000; anti-rabbit Bax, Abcam, San Francisco, USA, diluted 1:1,000; anti-mouse Caspase-3, Abcam, San Francisco, USA, diluted 1:500; and anti-rabbit β-actin, Santa Cruz, CA, USA, diluted 1:5,000) overnight at 4 °C. The β-actin protein was used as a loading control. Then, the membranes were incubated with the appropriate HRP-conjugated secondary antibodies. The bands were visualized with an enhanced chemiluminescence reagent (Thermo Fisher Scientific, Waltham, USA) and subsequently scanned and analysed with an image analyser (Bio-Rad, California, USA). The intensity of the protein bands was normalized to β-actin.

### Quantitative real-time polymerase chain reaction (qRT-PCR)

The levels of the JAK2, STAT3, Bcl-2, Bax and Caspase-3 mRNAs in the hippocampus were detected using qRT-PCR. Total RNA was isolated from the rat hippocampus with Trizol reagent (Applied Biosystems, Waltham, USA) according to a standard protocol. The total RNA concentration was determined using a Q3000 micro-volume spectrophotometer (Quawell Technology, San Jose, USA), and RNA quality was measured using 1% agarose gel electrophoresis. First-strand cDNA was synthesized using a RevertAid First Strand cDNA Synthesis Kit (Thermo Fisher Scientific, Waltham, USA) on a C1000 Touch^TM^ Thermal Cycler (Bio-Rad, California, USA) according to the manufacturer’s instructions. Primers were designed based on published mRNA sequences using Primer 3 primer selection software, and then synthesized by a professional biotechnology company (Sangon Biotech Co., Ltd., Shanghai, China). PCR was used to amplify the cDNA with a Power SYBR^®^ Green PCR Master Mix kit (Applied Biosystems, Waltham, USA) in a total volume of 20 μL on a CFX96 Real-time PCR System (Bio-Rad, California, USA) with the following cycling parameters: 95 °C for 10 min; 40 cycles of 95 °C for 15 s and 55 °C for 1 min; followed by 65 °C for 5 s and 95 °C for 15 s. The amplification reactions were performed in triplicate. Melting curves were obtained after qRT-PCR to demonstrate the specific amplification of the gene of interest. A standard curve assay was performed to determine the amplification efficiency of the primers used in this study (Supplementary Figs 1–5 and Table 1). The relative differences in expression between groups were calculated using the threshold cycle values normalized to GAPDH, which has been shown to exhibit stable expression between conditions (as shown in Supplementary Fig. 6). The relative differences between the control and the other groups were calculated as relative increases by setting the control to 100%. Table 1 shows the primer sequences.

**Table 1:** Primer sequences used for the PCR analysis.

Gene		Sequences
GAPDH	forward reverse	5′-CCATTCTTCCACCTTTGAT-3′ 5′-TGGTCCAGGGTTTCTTACT-3′
JAK2	forward reverse	5′-TATGCTCCCGAATCCTTGAC-3′ 5′-TCTGCCCTTGTTTATCATTGC-3′
STAT3	forward reverse	5′-AAACTGCTTGCCTTGACCAC-3′ 5′-CGCCTTGCCTTCCTAAATAC-3′
Bcl-2	forward reverse	5′-GAGCGTCAACAGGGAGATGT-3′ 5′-CAGCCAGGAGAAATCAAACAG-3′
Bax	forward reverse	5′-AAGAAGCTGAGCGAGTGTCT-3′ 5′-CAAAGATGGTCACTGTCTGC-3′
Caspase-3	forward reverse	5′-AGCTGGACTGCGGTATTGAG-3′ 5′-GGGTGCGGTAGAGTAAGCAT-3′

### Statistical analysis

All data were analysed using SPSS 17.0 software and presented as the means ± standard errors of the means (SEM). Statistical significance was assessed using one-way analysis of variance (ANOVA) followed by Bonferroni’s *post hoc* test for multiple comparisons. A *P-*value < 0.05 was considered statistically significant.

## Electronic supplementary material

Supplemental Files
