# Supplementary material for: Xiaoyaosan exerts anxiolytic-like effects by down-regulating the TNF-α/JAK2-STAT3 pathway in the rat hippocampus
Source: Sci Rep. 2017 Mar 23;7:353. doi: 10.1038/s41598-017-00496-y (PMC5428435; doi:10.1038/s41598-017-00496-y)
Supplement: Supplementary file 1 — Supplemental Files [file 41598_2017_496_MOESM1_ESM.doc]

**Supplemental Files for:**

**Xiaoyaosan exerts anxiolytic-like effects by down-regulating the TNF-α/JAK2-STAT3 pathway in the rat hippocampus**

Xiao-Juan Li^+^, Qing-Yu Ma^+^, You-Ming Jiang, Xiao-Hui Bai, Zhi-Yi Yan, Qun Liu, Qiu-Xia Pan, Yue-Yun Liu and Jia-Xu Chen^*^

**Institutional address:** School of Basic Medical Science, Beijing University of Chinese Medicine, No. 11 North Third Ring Road Chaoyang District, Beijing 100029, China

**Email addresses:** Xiao-Juan Li: 15652608965@163.com;

Qing-Yu Ma: 20140941026@bucm.edu.cn;

You-Ming Jiang: 115636520@qq.com

Xiao-Hui Bai: baixhzmz@163.com;

Zhi-Yi Yan: 15010190928@163.com;

Qun Liu: agnes57459@hotmail.com;

Qiu-Xia Pan: pqx1126@sina.com;

Yue-Yun Liu: chloelou@126.com;

Jia-Xu Chen: chenjiaxu@hotmail.com

***Corresponding author:** Email: chenjiaxu@hotmail.com

**^+^These authors contributed equally to this work**

**Supplement table 1** Amplification efficiencies in real-time quantitative PCR

| Gene | Amplification efficiency (%) | R^2^ | Slope |
| --- | --- | --- | --- |
| GAPDH | 95.0-98.4 | 0.995-0.999 | 3.360-3.449 |
| JAK2 | 97.1 | 0.995 | 3.394 |
| STAT3 | 97.9 | 0.999 | 3.373 |
| Bcl-2 | 96.5 | 0.991 | 3.408 |
| Bax | 95.4 | 0.993 | 3.438 |
| Caspase-3 | 97.3 | 0.994 | 3.388 |

**Supplement figure 1**


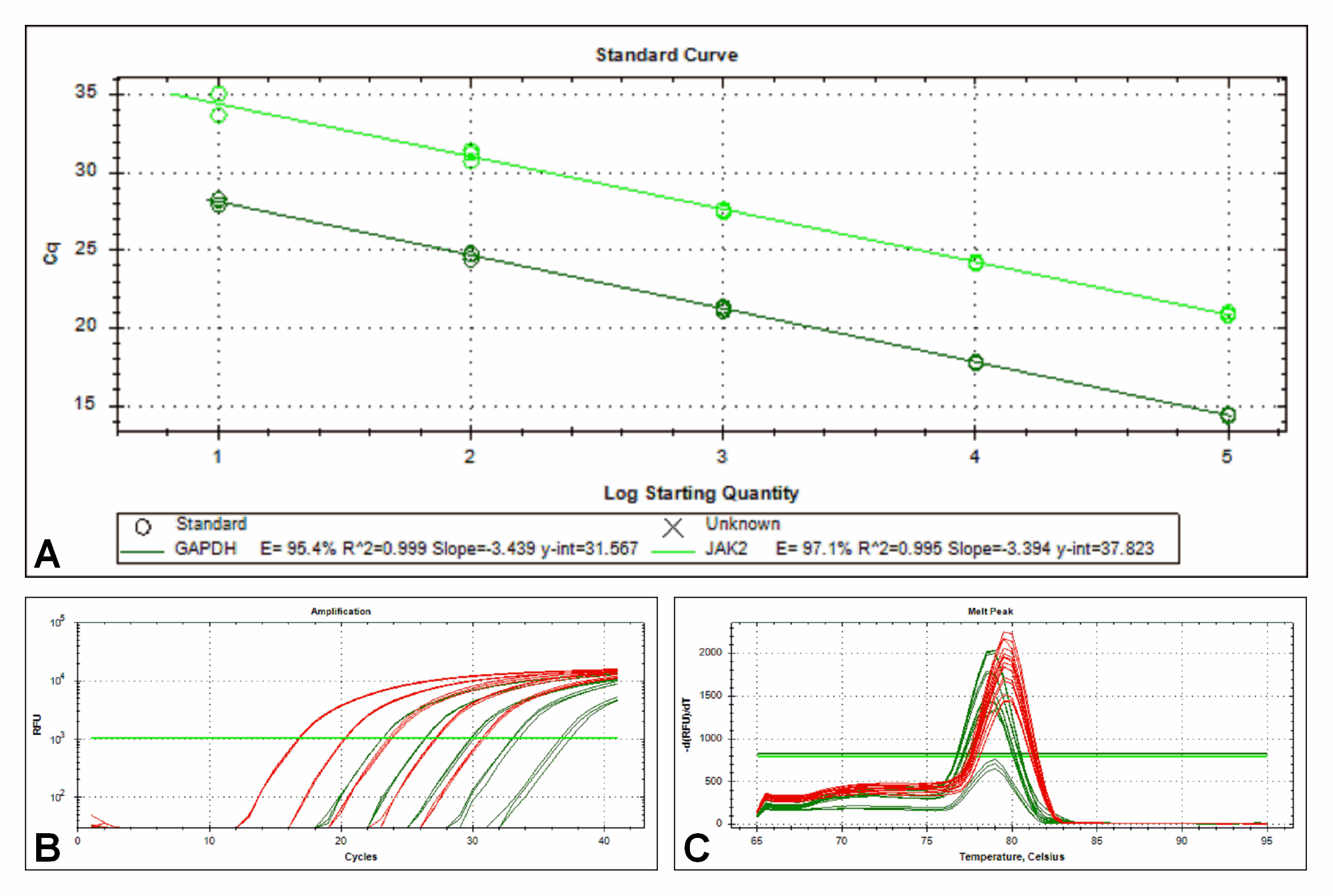


**Supplement figure 1.** Amplification efficiencies of GAPDH and JAK2 in real-time quantitative PCR. A: Standard curve. B: Amplification curve (log scale). C: Melting curve.

**Supplement figure 2**


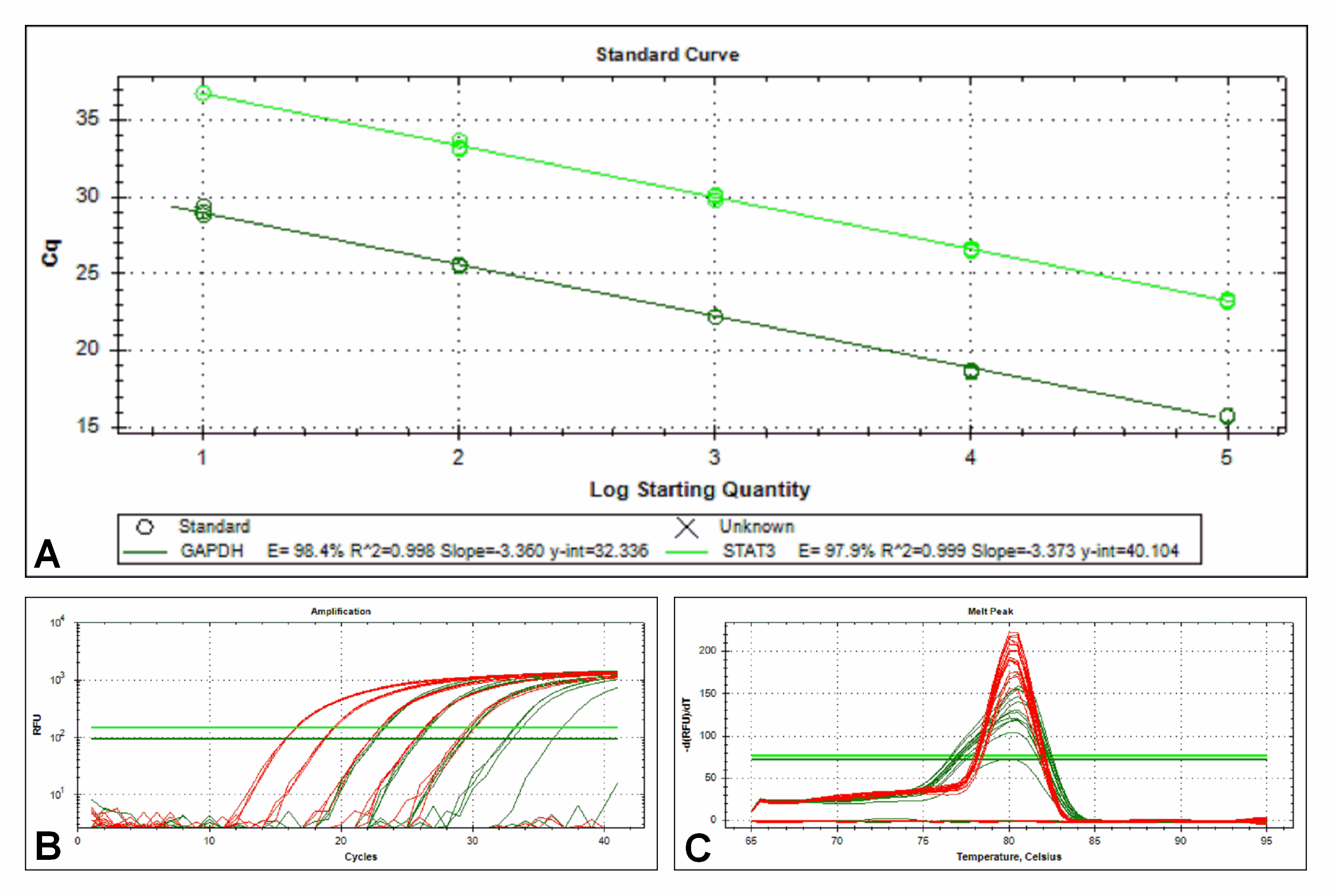


**Supplement figure 2.** Amplification efficiencies of GAPDH and STAT3 in real-time quantitative PCR. A: Standard curve. B: Amplification curve (log scale). C: Melting curve.

**Supplement figure 3**


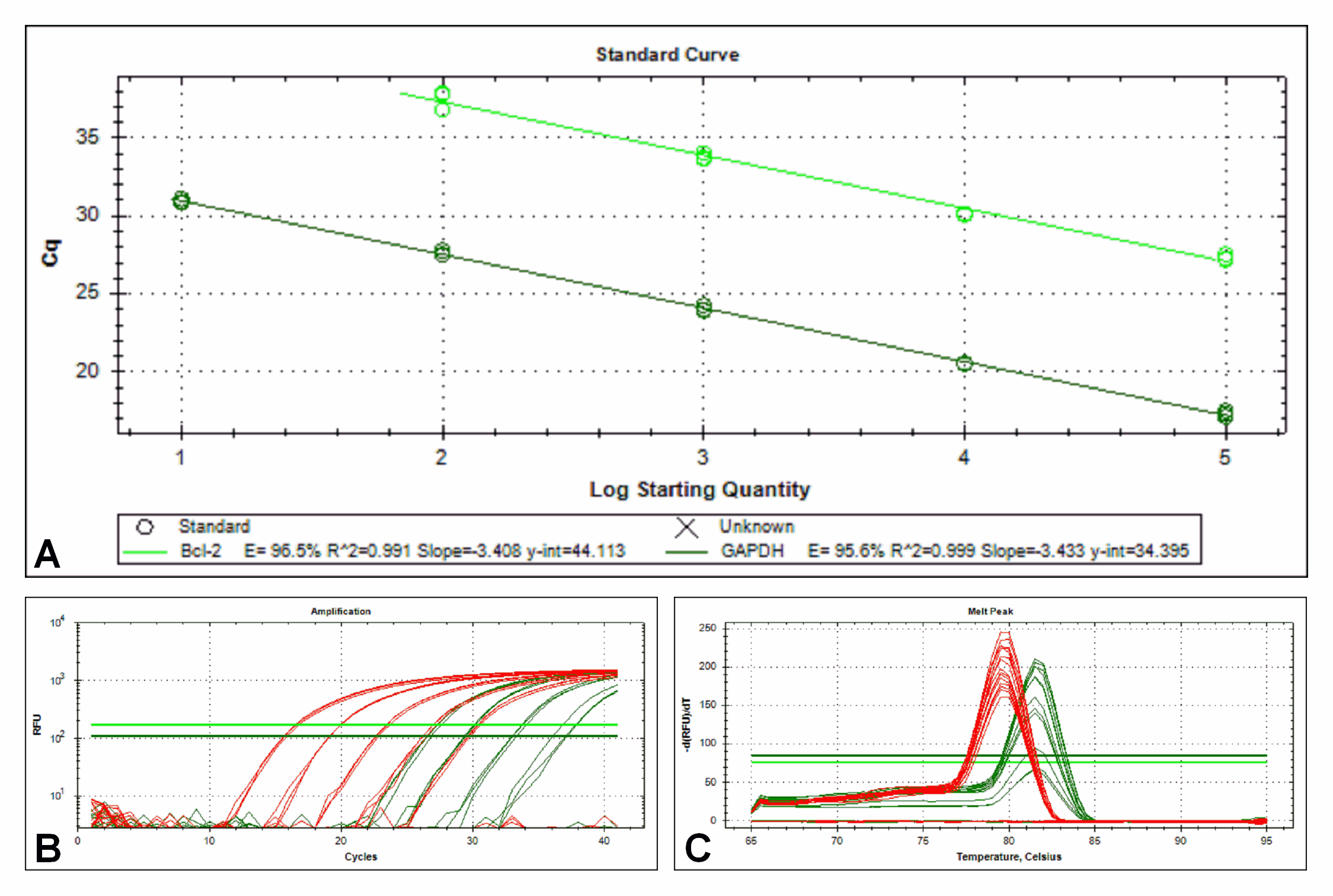


**Supplement figure 3.** Amplification efficiencies of GAPDH and Bcl-2 in real-time quantitative PCR. A: Standard curve. B: Amplification curve (log scale). C: Melting curve.

**Supplement figure 4**


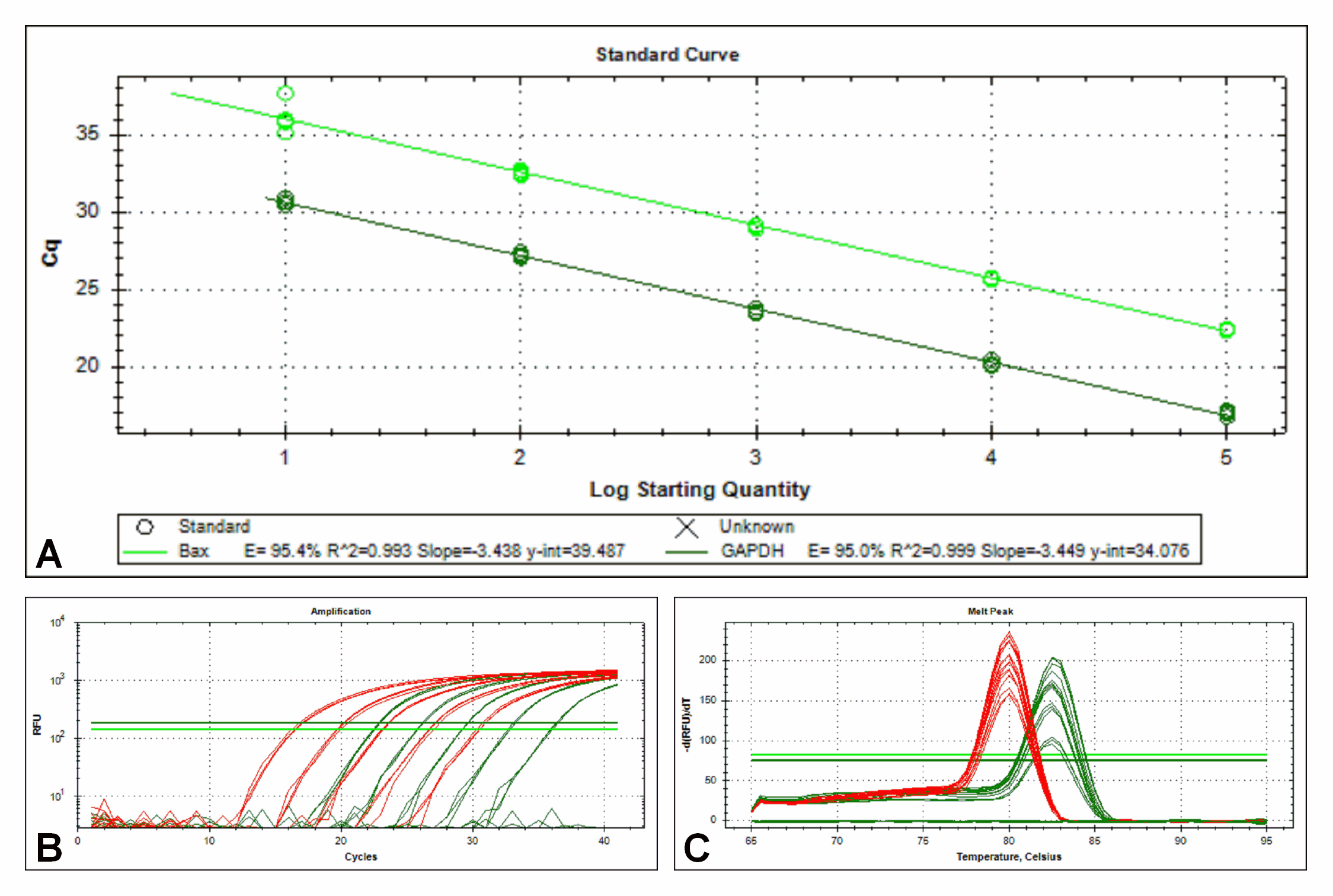


**Supplement figure 4.** Amplification efficiencies of GAPDH and Bax in real-time quantitative PCR. A: Standard curve. B: Amplification curve (log scale). C: Melting curve.

**Supplement figure 5**


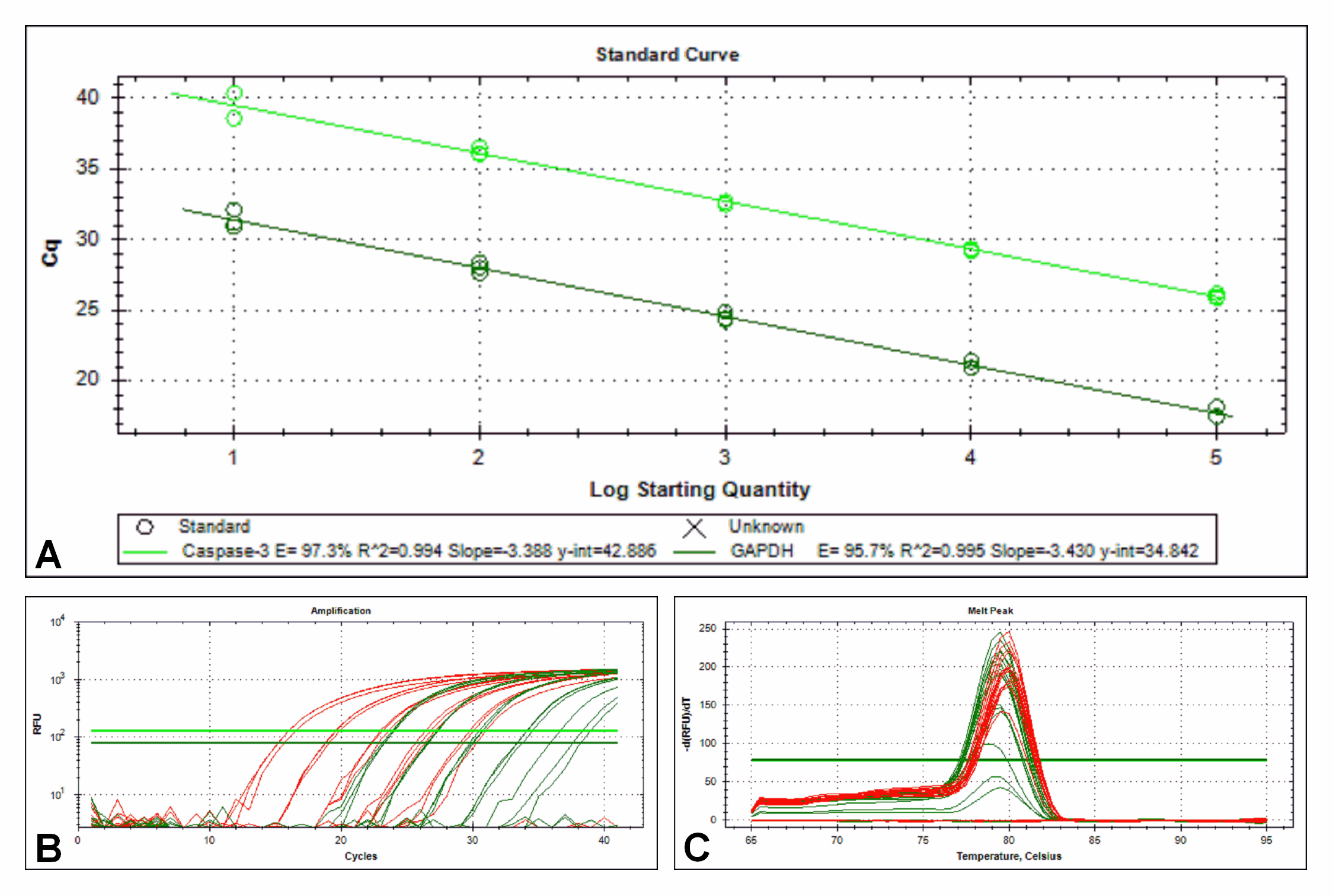


**Supplement figure 5.** Amplification efficiencies of GAPDH and Caspase-3 in real-time quantitative PCR. A: Standard curve. B: Amplification curve (log scale). C: Melting curve.

**Supplement figure 6**


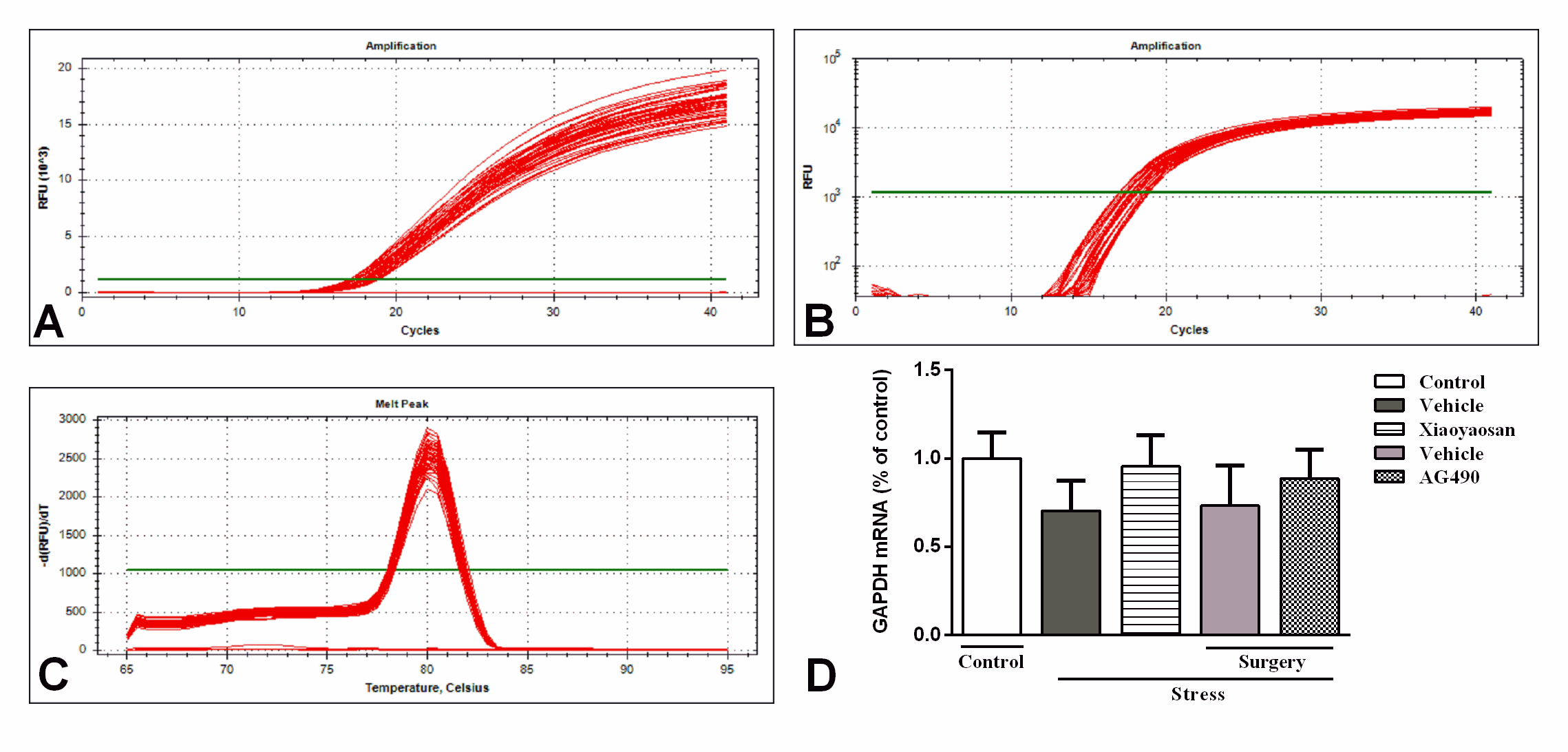


**Supplement figure 6.** Relative differences in GAPDH expression between the control and the other groups (the control was set to 100%). A: Amplification curve (linear scale). B: Amplification curve (log scale). C: Melting curve. D: GAPDH mRNA expression in the rat hippocampus. Values are presented as the means ± SEM from 5 rats in each group.

**Supplement figure 7**


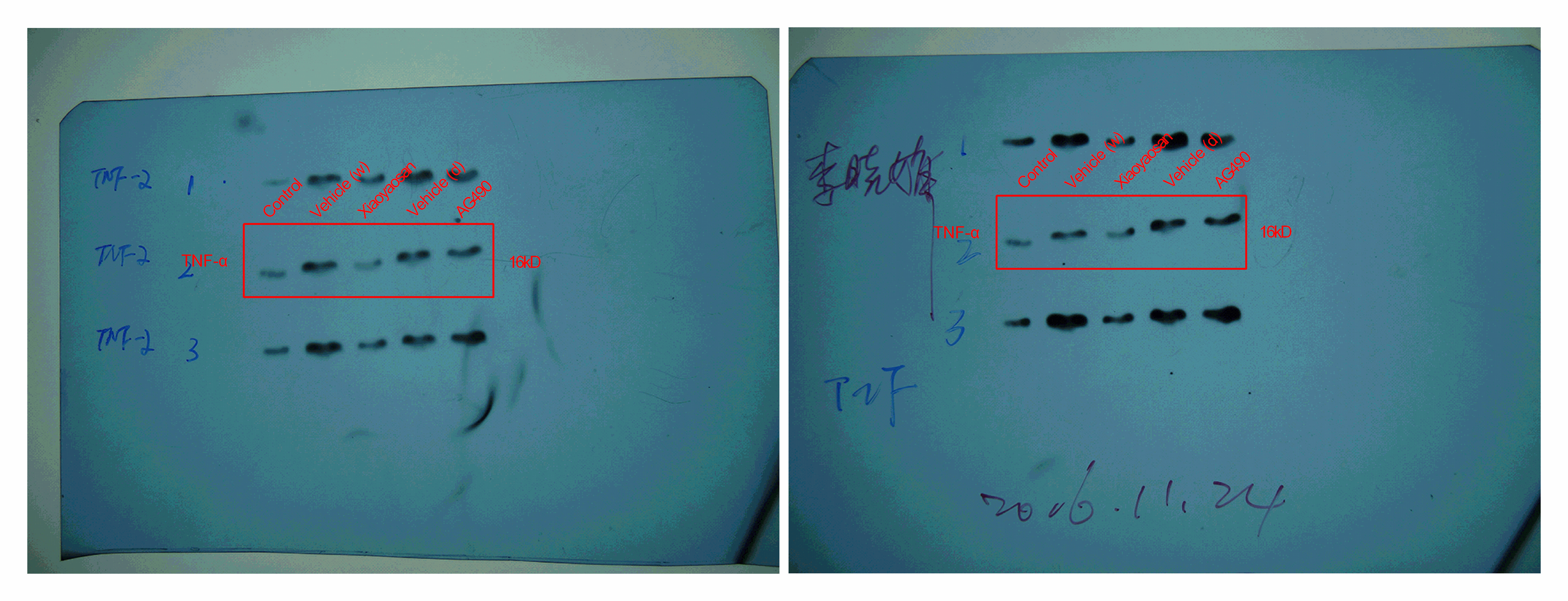


**Supplement figure 7.** The Kodak film displayed the multiple exposures of Fig 4B.

**Supplement figure 8**


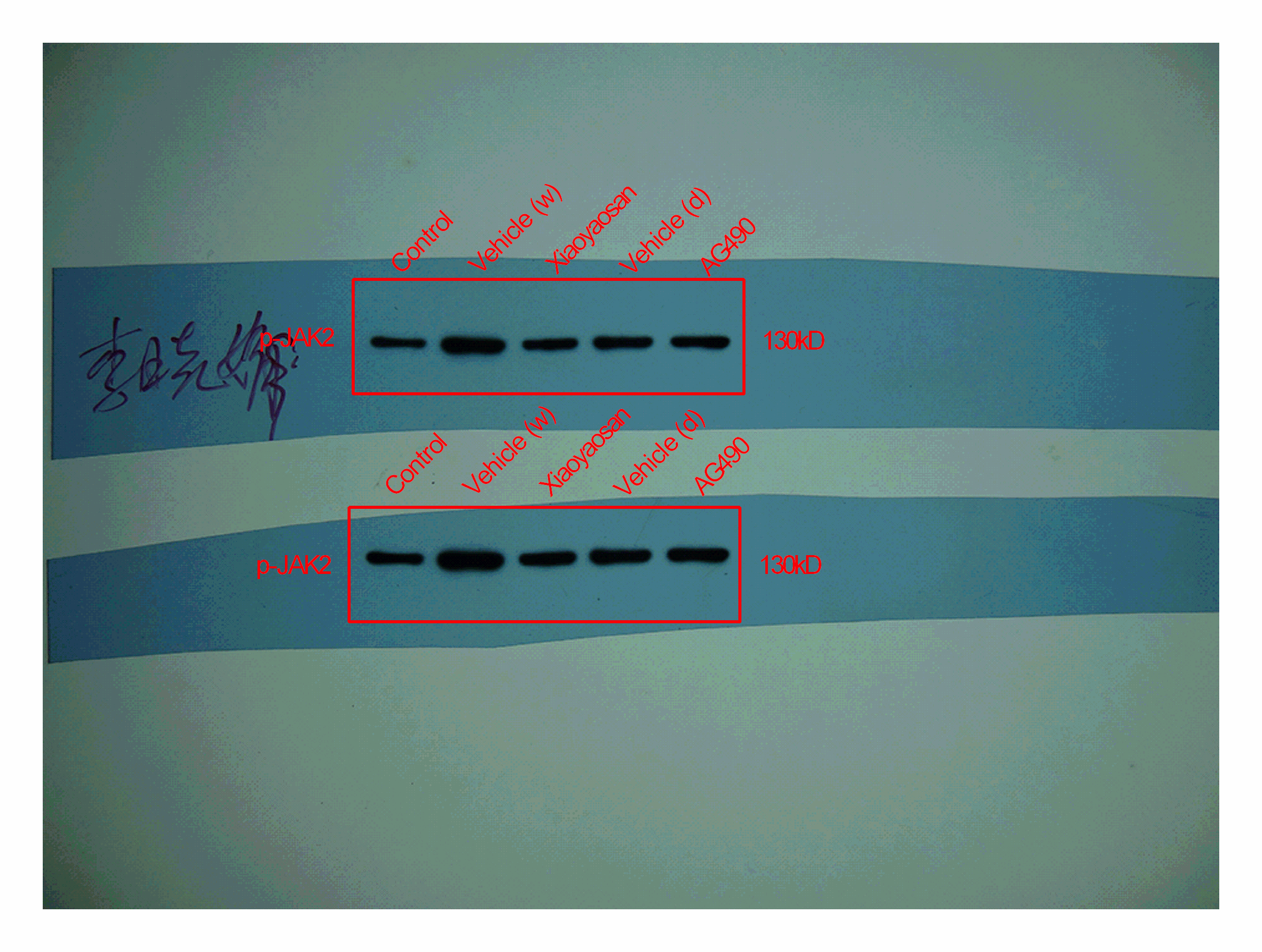


**Supplement figure 8.** The Kodak film displayed the multiple exposures of Fig 5A.

**Supplement figure 9**


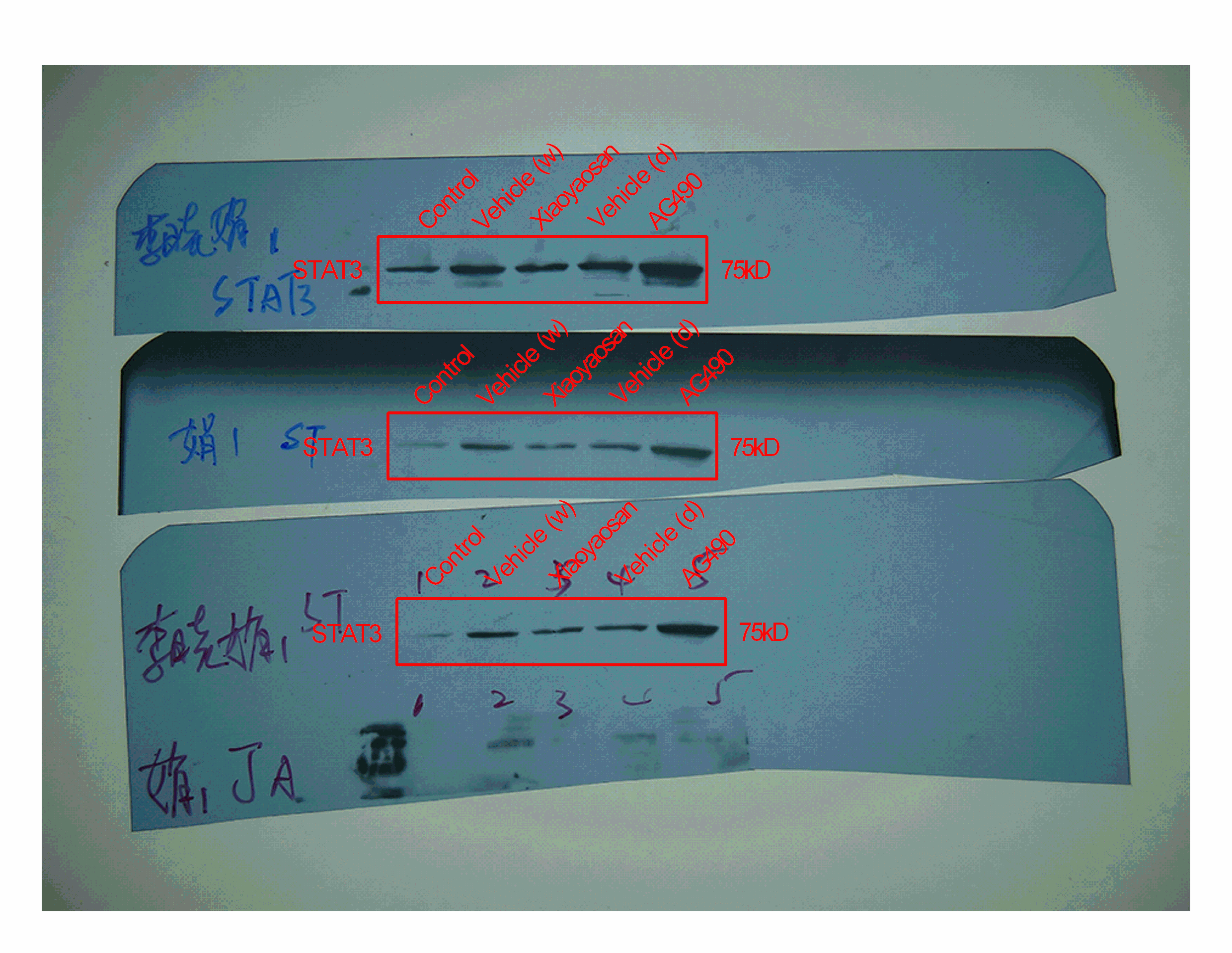


**Supplement figure 9.** The Kodak film displayed the multiple exposures of Fig 5C.

**Supplement figure 10**


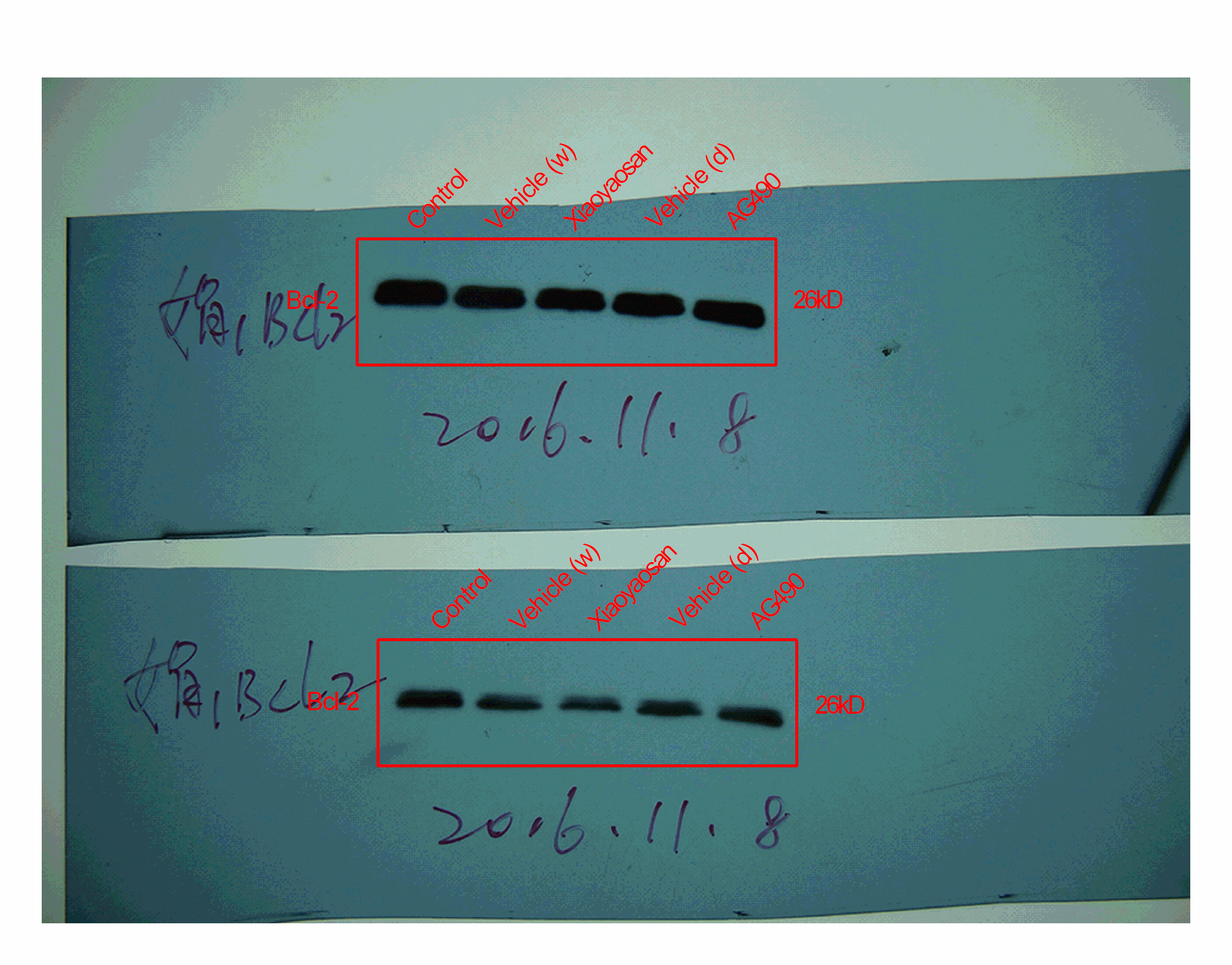


**Supplement figure 10.** The Kodak film displayed the multiple exposures of Fig 6A.

**Supplement figure 11**


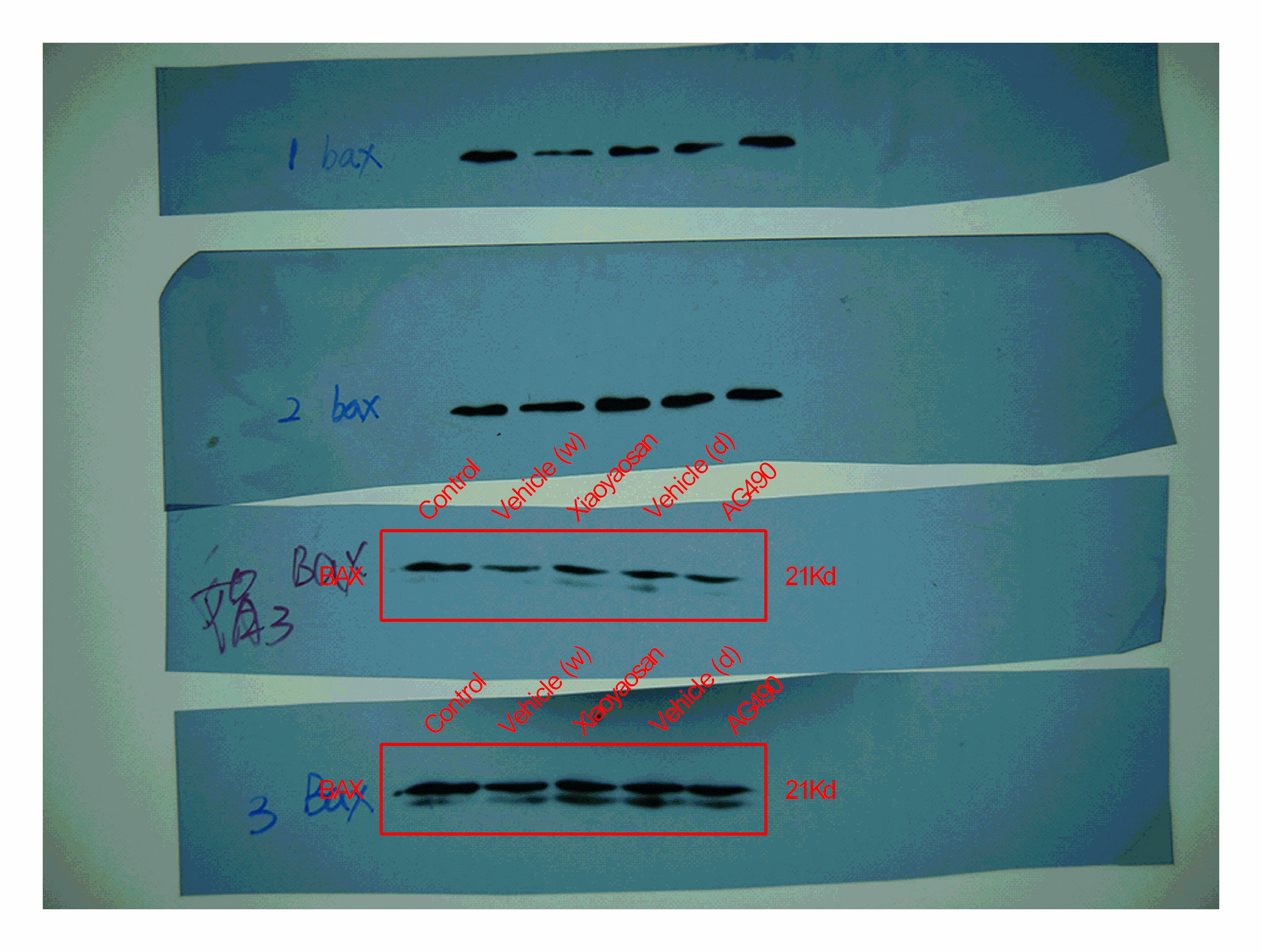


**Supplement figure 11.** The Kodak film displayed the multiple exposures of Fig 6B.

**Supplement figure 12**


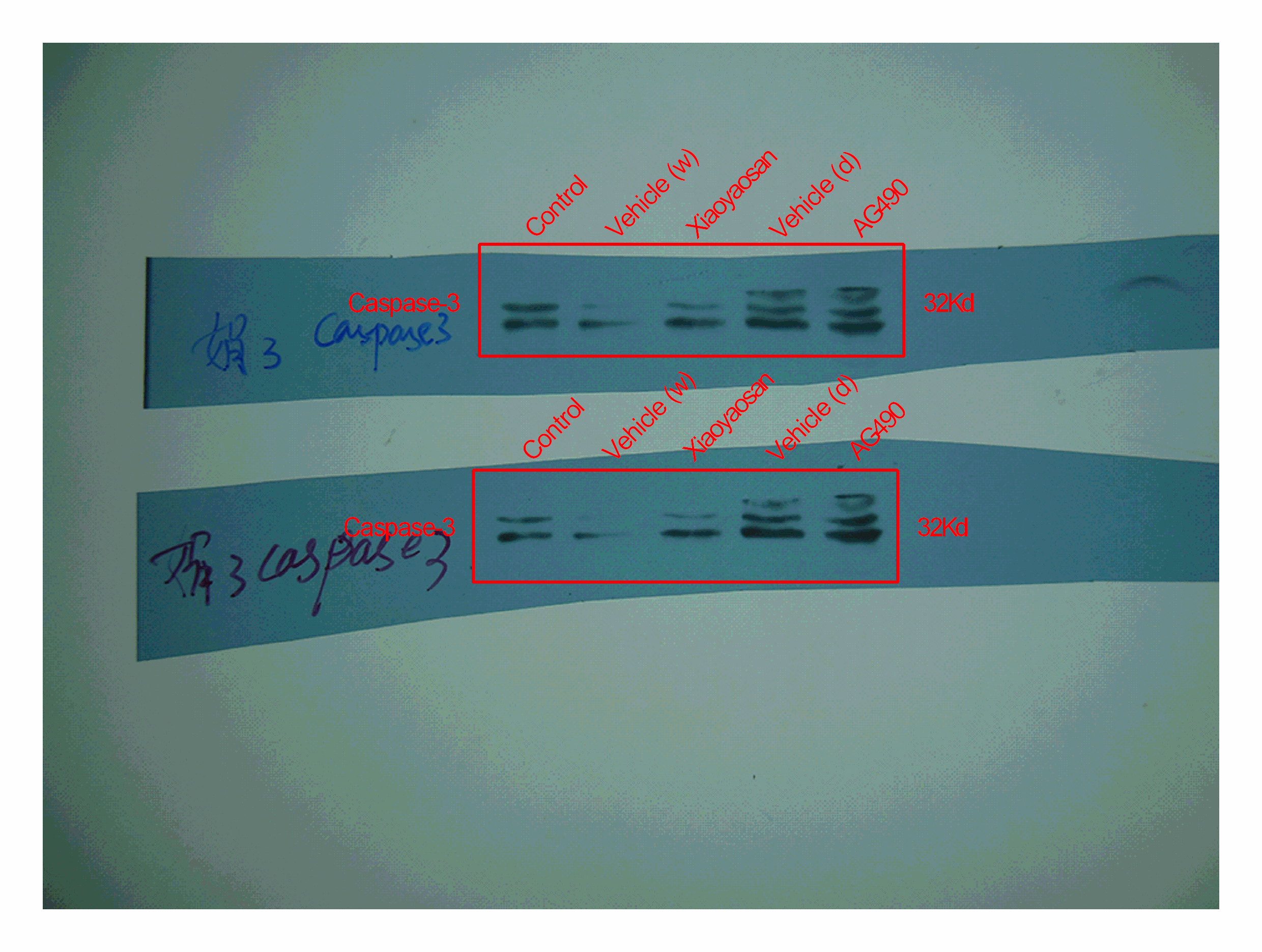


**Supplement figure 12.** The Kodak film displayed the multiple exposures of Fig 6C.
